# Substrate-field-modulated remote-van der Waals hybrid epitaxy in transition metal dichalcogenide heterostructures

**DOI:** 10.1186/s40580-026-00542-4

**Published:** 2026-03-28

**Authors:** Lia Saptini Handriani, Suhee Jang, Yelim Kim, Hyuncheol Yun, Dae Yeop Jeong, Hyeonsu Park, Zhe Gao, Jae-il Jang, Won Il Park

**Affiliations:** Division of Materials Science and Engineering, Hanyang University, Seoul, 04763 Republic of Korea

**Keywords:** Transition metal dichalcogenides (TMDCs), Vertical heterostructures, Remote epitaxy, Van der Waals epitaxy, Remote–vdW hybrid epitaxy, Two-dimensional materials, MOCVD growth, Nucleation kinetics

## Abstract

Two-dimensional (2D) transition-metal dichalcogenide (TMDC) heterostructures are promising for next-generation optoelectronics, yet the mechanisms controlling their vertical heteroepitaxy remain poorly understood. Here, we systematically investigate metal–organic chemical vapor deposition growth of MoS_2_/WS_2_ and WS_2_/MoS_2_ vertical heterostructures across varying interlayer thicknesses (monolayer to multilayer) and substrates (Si, SiO_2_ and c-sapphire). We identify a substrate-field-modulated “remote–van der Waals (vdW) hybrid epitaxy” regime, in which vertical overgrowth is confined to a narrow thickness window (~ 1–3 layers), with nucleation density strongly influenced by substrate polarity and defect chemistry. High-resolution STEM reveals that, in the regions where vertical growth occurs, the in-plane crystallographic registry is primarily governed by vdW coupling to the 2D template, yielding a highly preferred single-orientation registry across the examined regions for both stacking orders. This dual-control mechanism decouples growth propensity from epitaxial alignment, providing a scalable framework for synthesizing high-quality 2D vertical heterostructures with precisely engineered interfaces.

**Graphical abstract:**

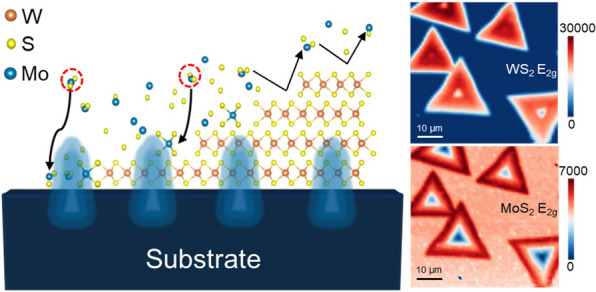

**Supplementary Information:**

The online version contains supplementary material available at 10.1186/s40580-026-00542-4.

## Introduction

Atomically thin two-dimensional (2D) semiconductor materials, such as transition-metal dichalcogenides (TMDCs), have attracted significant attention owing to their tunable bandgaps, strong light-matter interactions, and compatibility with heterogeneous integration. Despite their reduced dimensionality, these materials support rich electronic and optical phenomena, making them highly promising for applications in optoelectronics, photonics, spin/valleytronics, and quantum technologies [1–9]. Integrating different TMDC layers into 2D heterostructures (HSs) further extends this versatility, enabling interlayer excitons and charge transfer [10–12], enhanced light absorption, and gate-tunable correlated and topological phases that do not exist in the constituent monolayers [13–16].

TMDC HSs are typically classified into vertical HSs, formed by stacking distinct TMDC layers, and lateral HSs, formed by seamless in-plane junctions between different TMDC domains. Mechanical exfoliation offers high-quality single-crystal layers for fundamental studies such as unconventional superconductivity and topological insulator states [17, 18]. However, it remains intrinsically difficult to scale and does not readily provide wafer-level control over layer thickness, spatial placement, and crystallographic orientation.

In contrast, chemical vapor deposition (CVD), including metal–organic CVD (MOCVD), has emerged as a scalable route to TMDC HSs with wafer-scale coverage and thickness control [19]. By tuning precursor concentration, temperature, and pressure, CVD growth can be steered towards either lateral or vertical HS formation. Experiments indicate that nucleation, adatom diffusion, and crystallinity are strongly influenced by interfacial defects and surface compatibility [20–27]. Despite these advances, the mechanisms governing vertical 2D-on-2D heteroepitaxy—particularly between atomically smooth, chemically inert basal planes—remain insufficiently understood.

Extensive studies have shown that atomically thin materials only partially screen their substrates and remain effectively “transparent” to the underlying surface. As a result, hydrophilicity, electric double-layer structure, charge doping, and even lattice potential can still be strongly influenced by the underlying support, even through monolayer graphene or other 2D sheets [28–32]. This partial transparency indicates that the presence of a 2D interlayer does not fully decouple a material from its substrate and that substrate-induced fields and chemistry can still be communicated through atomically thin layers. As the intermediate layer becomes thicker, however, the substrate influence is progressively screened. In conventional van der Waals (vdW) epitaxy, the overlayer typically grows on thick, dangling-bond–free vdW surfaces [33, 34]. Such chemically passive surfaces allow the overlayer to be guided mainly by weak vdW interactions, enabling tolerance to substantial lattice mismatch. However, when the interlayer is reduced to an atomically thin 2D sheet on a polar or ionic substrate, the substrate potential can penetrate the spacer and imprint its lattice on the overgrown film, leading to the notion of remote epitaxy [35–39].

To date, vdW and remote epitaxy concepts have been explored predominantly for 3D or quasi-3D heteroepitaxial films grown on 2D layer-coated substrates, where the emphasis is on enabling lattice-mismatched integration and mechanical release. In contrast, how substrate properties and interlayer thickness jointly control nucleation, growth kinetics and crystallographic registry in fully 2D–2D vertical HSs remains much less systematically explored.

In this work, we address these open questions by systematically examining vertical crystal growth in a fully 2D–2D system using MOCVD. We grow MoS_2_/WS_2_ and WS_2_/MoS_2_ vertical heterostructures on Si, SiO_2_ and c-sapphire, examining overgrowth across various 2D interlayer thicknesses (from monolayer to multilayer). From these observations, we identify a dual-control regime, which we term substrate-field-modulated remote–van der Waals hybrid epitaxy (remote–vdW hybrid epitaxy). In this regime, the substrate’s electrostatic field and defect chemistry govern the nucleation density and the active thickness window (1–3 layers) for overgrowth, whereas the in-plane crystallographic registry is dictated primarily by the 2D template through van der Waals coupling. This hybrid mechanism—bridging remote and van der Waals epitaxy—enables the synthesis of single-orientation 2D vertical heterostructures with a highly preferred single-orientation registry and no detectable mirror-twin signatures within the representative sampled areas. This provides a practical, scalable framework for the large-area growth of high-quality TMDC heterostructures with engineered interfaces.

## Methods

### Synthesis of MoS_2_/WS_2_ vertical heterostructures

#### *Growth of WS*_*2*_* templates on SiO*_*2*_*/Si*

Triangular WS_2_ flakes were synthesized on SiO_2_/Si substrates using a custom-built vertical cold-wall MOCVD reactor equipped with a rotating graphite susceptor. Tungsten hexacarbonyl (W(CO)_6_, WHC) and diethyl sulfide ((C_2_H_5_)_2_S, DES) served as tungsten and sulfur precursors, respectively. The solid WHC and liquid DES were maintained in temperature-controlled bubblers at 30 °C and 55 °C and delivered into the quartz-tube reactor using Ar carrier gas [25, 26].

To promote large-domain growth, a NaCl-assisted strategy was employed by placing a circular crucible containing 0.6 g of NaCl (Sigma-Aldrich) at the center of the susceptor. Growth was conducted at 650 °C and 5 Torr for 20 min, with WHC and DES introduced at equal flow rates (6 sccm each) under a background flow of 140 sccm Ar and 10 sccm H_2_. The as-grown WS_2_ flakes were subsequently used as templates for MoS_2_ overgrowth in the MoS_2_/WS_2_ configuration.

### *MoS*_*2*_* overgrowth on WS*_*2*_*/SiO*_*2*_

MoS_2_/WS_2_ vertical heterostructures were obtained by a second MOCVD step in the same reactor. Molybdenum hexacarbonyl (Mo(CO)_6_) and DES were used as Mo and S precursors, respectively. Mo(CO)_6_ was held at 30 °C in a temperature-controlled bubbler and delivered by Ar carrier gas, while DES was supplied from a liquid bubbler. The reactor temperature, pressure and carrier-gas flows were kept identical to those used for WS_2_ template growth (650 °C, 5 Torr, 140 sccm Ar, 10 sccm H_2_). Crucially, no NaCl or other transport agents were introduced during this second step to ensure that the MoS_2_ overgrowth was governed solely by the template-substrate interaction.

The Mo(CO)_6_ and DES flow rates were adjusted to maintain a sulfur-rich environment, and growth times in the range of 30–60 min were used to form MoS_2_ overlayers on the WS_2_/SiO_2_ templates. These samples, which contain WS_2_ thickness gradients (*n* = 0 to multilayer), were used for the thickness- and time-dependent MoS_2_ overgrowth studies presented in Sect. 3.1 of the main text.

### Synthesis of WS_2_/MoS_2_ vertical heterostructures

#### *Preparation of thickness-graded MoS*_*2*_* templates by Au-assisted exfoliation*

For the reciprocal WS_2_/MoS_2_ configuration, thickness-graded MoS_2_ templates were prepared by gold-assisted mechanical exfoliation. A metal adhesion stack of Ti/Au (2 nm/15 nm) was deposited onto p-type Si substrates with a 300 nm SiO_2_ layer using an electron-beam evaporator (Woosin CryoVac) at a deposition rate of 0.5 Å·s⁻^1^. Commercial bulk MoS_2_ crystals (HQ Graphene; 2D Semiconductors) were exfoliated using adhesive tape and brought into conformal contact with the Au-coated substrates.

To enhance the yield of large-area monolayers, the substrate was heated to 130–150 °C for ~ 90 s during contact, followed by a ~ 60 s cooling period before tape removal. The underlying Ti/Au layer was then removed using an aqueous KI/I_2_ etchant, and the samples were annealed at 350 °C for 2 h in H_2_ atmosphere to remove organic residues and improve interface cleanliness. This procedure produced MoS_2_ flakes spanning monolayer to multilayer thicknesses (n = 1–multilayer), which served as templates for reciprocal WS_2_ overgrowth on SiO_2_/Si and for substrate-comparison experiments on Si and c-sapphire.

### *WS*_*2*_* overgrowth on exfoliated MoS*_*2*_* templates*

WS_2_/MoS_2_ vertical heterostructures were grown in the same vertical MOCVD reactor used for WS_2_ template synthesis. WHC and DES were supplied from temperature-controlled bubblers (30 °C for WHC, 55 °C for DES) and introduced at equal flow rates of 6 sccm under 140 sccm Ar and 10 sccm H_2_ at 650 °C and 5 Torr. WS_2_ was deposited on the Au-assisted exfoliated MoS_2_ templates described above, which span monolayer to multilayer thicknesses.

Growth times in the range of 7–15 min were used to capture the early nucleation and vertical build-up of WS_2_ on MoS_2_, while longer growths were employed for the fully developed WS_2_/MoS_2_ stacks analyzed in the main text. For substrate-comparison experiments, identically prepared MoS_2_ templates were transferred onto Si, SiO_2_ and c-sapphire and subjected to the same WS_2_ growth conditions, allowing direct assessment of substrate-field effects at fixed MoS_2_ thickness.

### Optical and structural characterization

The morphology and layer contrast of MOCVD-grown WS_2_ templates, MoS_2_/WS_2_ heterostructures and WS_2_/MoS_2_ stacks were first examined by optical microscopy (Olympus BX51). Atomic force microscopy (AFM) was used to determine step heights and verify the layer number of exfoliated MoS_2_ and MOCVD-grown WS_2_ flakes, as well as to compare the apparent MoS_2_ thickness on WS_2_ versus SiO_2_ (Figs. S3 and S4).

Room-temperature Raman and photoluminescence (PL) measurements were performed using a micro-Raman system (Dongwoo Optron) equipped with a 532 nm excitation laser and 50 × /100 × objective lenses. Raman and PL mapping was carried out on predefined grids to resolve thickness-dependent growth windows and excitonic behavior. Spectral datasets were processed using baseline subtraction followed by multi-Gaussian peak fitting, as described in Supplementary Note 1, to deconvolute overlapping MoS_2_ and WS_2_ modes and obtain reliable intensity and peak-position maps.

For structural analysis at the nanoscale, high-resolution transmission electron microscopy (HR-TEM, JEOL JEM-F200, 200 kV) was used to assess lattice crystallinity and acquire selected-area electron diffraction (SAED) patterns over micrometer-scale regions. Atomic-scale interface structure, stacking registry and local moiré superlattices were examined by spherical-aberration-corrected high-angle annular dark-field scanning TEM (HAADF-STEM, JEOL JEM-ARM200F, 80 kV). Cross-sectional lamellae were prepared using a focused ion beam (FIB, FEI Helios 650) to directly visualize the vertical heterostructure geometry, confirm the presence or absence of WS_2_ overgrowth on MoS_2_ templates of different thicknesses, and perform elemental mapping via energy-dispersive X-ray spectroscopy (EDS).

## Results and discussion

### Thickness-selective and time-dependent MoS_2_ overgrowth on WS_2_/SiO_2_

**Fig. 1:**
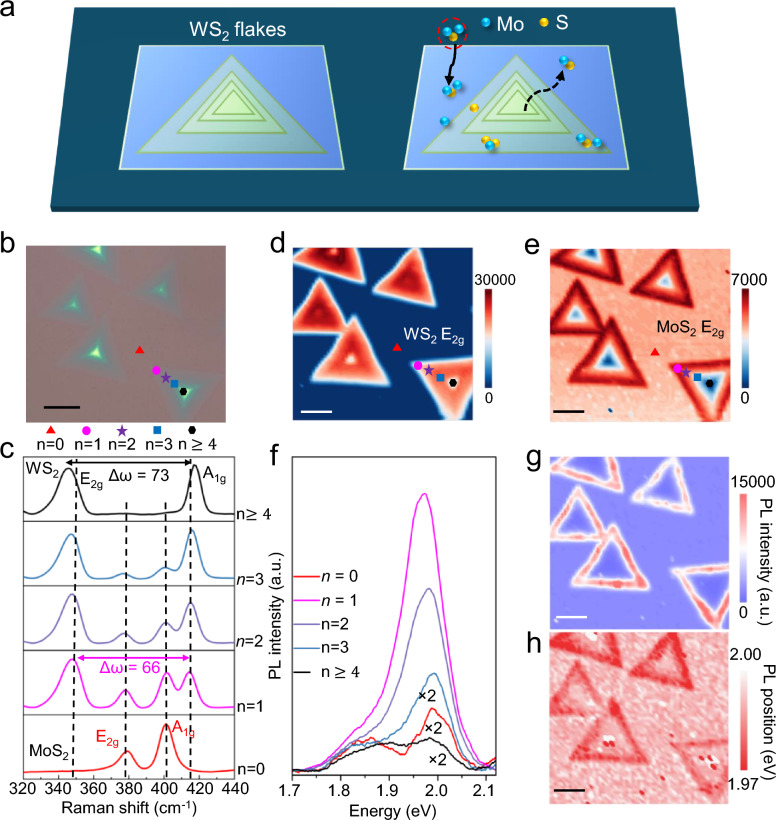
Thickness-dependent heterostructure growth of MoS_2_ on WS_2_ templates. **a**, Schematic illustration of the vertical MOCVD growth of MoS_2_ on a triangular WS_2_ flake supported on SiO_2_/Si. **b**, Optical microscopy image of a representative WS_2_ flake showing contrast variations corresponding to different layer thicknesses (*n*). **c**, Representative Raman spectra extracted from the marked spots in (b), showing distinct MoS_2_ signatures on bare SiO_2_ (*n* = 0) and thin WS_2_ (*n* = 1–3), but absent signals on multilayer WS_2_ (*n* ≥ 4). **d**, **e**, Raman intensity maps of the WS_2_ E_2g_ (template) and MoS_2_ E_2g_ (overlayer) modes, respectively, visualizing the confinement of vertical growth to the atomically thin regions. **f**–**h**, Corresponding PL spectra (f), integrated intensity map (g), and peak position map (h) of the A exciton, confirming strong MoS_2_ emission from the monolayer/bilayer regions and suppression on the multilayer center**.** Scale bars in b, d–e, and g–h: 10 μm

Triangular WS_2_ flakes were synthesized on SiO_2_/Si substrates in our vertical MOCVD reactor using NaCl as a growth promoter (Methods). The addition of NaCl facilitates the formation of volatile tungsten oxychlorides, which enhances precursor mass transport and reduces nucleation density [40]. This strategy significantly increased both the lateral size and the crystalline quality of the WS_2_ flakes, providing large, continuous domains necessary for spatially resolved heterostructure studies. These WS_2_ flakes were subsequently used as interlayers to investigate the growth behavior of MoS_2_, as illustrated in Fig. 1a. OM images (Fig. 1b) reveal triangular domains up to ~ 35 μm across with clear thickness contrast, providing a platform to examine overgrowth as a function of WS_2_ layer number *n*.

To quantify MoS_2_ overgrowth across a thickness-gradient flake, we processed the Raman mapping dataset (2,601 spectra across a 51 × 51 grid) using baseline subtraction followed by multi-Gaussian fitting (Supplementary Note 1). This approach enables robust deconvolution of overlapping features, particularly resolving the MoS_2_ A_1g_ (~ 401 cm^−1^) mode adjacent to the WS_2_ A_1g_ (~ 414 cm^−1^) peak under a spatially varying background. By more realistically separating the contributions of individual peaks, this multi-Gaussian analysis improves the reliability of both the Raman spectra and the resulting intensity maps.

After 60 min of MoS_2_ deposition, representative fitted spectra extracted from the marked locations in Fig. 1b are shown in Fig. 1c. The WS_2_ layer number *n* was assigned from the A_1g_–E_2g_ separation (Δω), which increases from ~ 66 cm⁻^1^ for monolayer WS_2_ (*n* = 1) to ~ 73 cm⁻^1^ for multilayer WS_2_ (*n* ≥ 4), consistent with prior reports [41]. On this WS_2_ thickness-graded template, MoS_2_ peaks are observed on bare SiO_2_ (*n* = 0) and on thin WS_2_ (*n* = 1–3). In contrast, multilayer WS_2_ (*n* ≥ 4) shows only WS_2_ modes, with no detectable MoS_2_ signal (Fig. 1c). The corresponding Raman intensity maps (Fig. 1d,e) visualize this thickness-dependent growth window; the WS_2_ E_2g_ map delineates the WS_2_ template, whereas the MoS_2_ E_2g_ map shows overgrowth confined to bare SiO_2_ and thin WS_2_, with negligible signal on multilayer regions.

PL provides a complementary probe of this thickness-dependent behavior. The spectra in Fig. 1f show that the excitonic emission near ~ 2.0 eV is strongest when MoS_2_ is deposited on monolayer WS_2_ (*n* = 1) and decreases for *n* = 2–3, whereas it is weaker on bare SiO_2_ (*n* = 0) and becomes minimal on multilayer WS_2_ (*n* ≥ 4). Consistently, the PL map integrated at ~ 2.0 eV (Fig. 1g) reproduces the same thickness-dependent intensity variation across the flake, while the peak-position map (Fig. 1h) remains comparatively uniform. These results indicate that MoS_2_ grown on thin WS_2_ experiences reduced non-radiative quenching and improved optical quality compared with MoS_2_ on bare SiO_2_ [42–44]. In line with this picture, atomic force microscopy (AFM) measurements (Supplementary Note 2) show that the apparent MoS_2_ thickness increases more rapidly on SiO_2_ than on bilayer WS_2_, consistent with slower vertical build-up on the chemically inert WS_2_ surface, as schematically summarized in Fig. S3c.

**Fig. 2:**
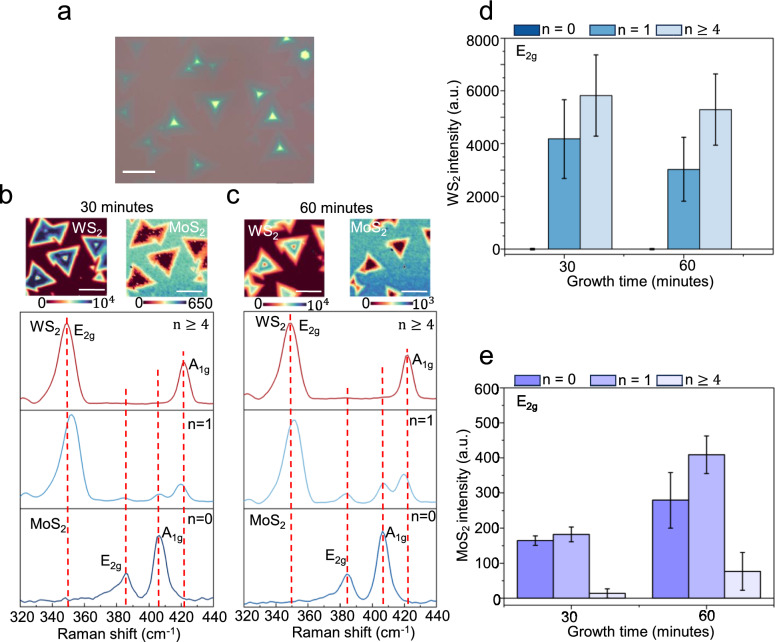
Time-resolved evolution of MoS_2_ vertical growth kinetics on WS_2_/SiO_2_. **a**–**c**, Raman intensity maps of the MoS_2_ E_2g_ mode acquired after different growth durations (30 min and 60 min), visualizing the progressive accumulation of MoS_2_ material. The maps reveal that overgrowth proceeds continuously on bare SiO_2_ (n = 0) and monolayer WS_2_ (n = 1) regions, whereas multilayer WS_2_ regions (n ≥ 4) show negligible MoS_2_ signal regardless of growth time. **d**, **e**, Statistical distributions of MoS_2_ Raman peak intensities extracted from the mapping data, quantifying the continued vertical growth on atomically thin templates versus the persistent suppression on thick WS_2_ layers over the 60-min period. Scale bars: 20 μm. Note that intensity values in non-growth regions were clamped to zero to remove baseline subtraction noise

To understand whether this thickness dependence is governed by growth kinetics, we performed time-resolved Raman measurements during MoS_2_ deposition on WS_2_/SiO_2_ (Fig. 2). Raman maps acquired after 30 and 60 min capture how the overgrowth evolves across regions of different WS_2_ thickness (Fig. 2b,c). As the growth time increases, the MoS_2_ E_2g_ and A_1g_ signals increase on bare SiO_2_ (*n* = 0) and on monolayer WS_2_ (*n* = 1), whereas the spectra collected on multilayer WS_2_ (*n* ≥ 4) retain only the WS_2_ modes with no appreciable MoS_2_ contribution. Statistics extracted from the maps (Fig. 2d,e) quantify this trend, showing continued growth of MoS_2_ on *n* = 0 and *n* = 1, but persistently negligible MoS_2_ intensity on multilayer WS_2_. We interpret this increase in Raman intensity as a progressive accumulation of MoS_2_, representing a composite process of lateral coverage expansion and subsequent vertical stacking. To differentiate between these factors, we corroborated the Raman trends with AFM topographic analysis (Supplementary Note 2), which independently verifies that the rising spectroscopic signal accompanies an actual increase in physical film height. These data indicate that, within the growth times probed here, material accumulation continues only on bare SiO_2_ and atomically thin WS_2_, while multilayer templates remain effectively inactive for MoS_2_ growth. This thickness-selective evolution suggests that the buried substrate can still influence the effective growth environment through an atomically thin WS_2_ interlayer, but this influence becomes strongly attenuated once the WS_2_ exceeds a few layers in thickness.

To quantify the physical length scale of this thickness-dependent selectivity, we evaluated the Debye screening length λ_D_ of the TMDC template at the growth temperature (650 °C). By considering the thermal activation of carriers and the specific out-of-plane dielectric constant of the TMDC lattice [45], the estimated λ_D_ ranges from 0.7 to 2.2 nm. This theoretical range matches the observed active window of ~ 1–3 layers (~ 0.65–1.95 nm), supporting the interpretation that vertical nucleation is governed by the penetration of the substrate's electrostatic field, which becomes effectively screened as the interlayer thickness exceeds a few Debye lengths (see Supplementary Note 3 for detailed calculations). Our observation of field penetration through monolayer WS_2_ is consistent with previous reports on the ‘dielectric transparency’ of 2D materials. Theoretical and experimental studies have shown that the screening capability of monolayer TMDCs is significantly reduced compared to their bulk counterparts due to the dimensional confinement of electric field lines [46, 47]. Consequently, the underlying substrate can exert a strong, uniform electrostatic influence through the monolayer—an effect that is fundamentally distinct from localized defect-mediated interactions.

### Substrate-dependent nucleation on atomically thin WS_2_ interlayers

**Fig. 3:**
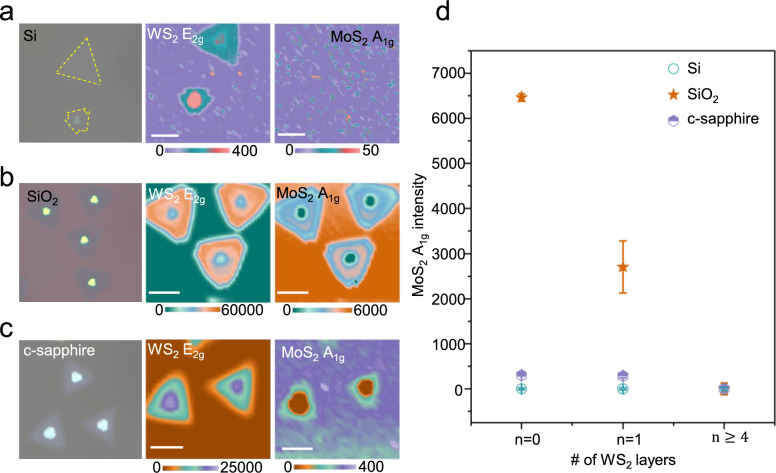
Substrate-dependent nucleation hierarchy of MoS_2_ on atomically thin WS_2_ interlayers. **a**–**c**, Representative Raman spectra and optical images comparing MoS_2_ overgrowth on monolayer WS_2_ (*n* = 1) supported by three different substrates: Si, SiO_2_ and c-sapphire. The data reveal a distinct growth hierarchy where nucleation is most abundant on SiO_2_, intermediate on sapphire, and negligible on Si. **d**, Statistical summary of MoS_2_ growth yield versus WS_2_ interlayer thickness (*n*). The trends confirm that the substrate influence is transmitted through thin interlayers (*n* = 1–3) to modulate growth, but is fully screened by thicker multilayers (*n* ≥ 4), leading to suppressed growth regardless of the substrate. Scale bars: 10 μm

To isolate the role of the underlying substrate at fixed interlayer thickness, we compared MoS_2_ overgrowth on monolayer WS_2_ (*n* = 1) supported on Si, SiO_2_ and c-sapphire (Fig. 3a–c). For the same WS_2_ interlayer, the MoS_2_ A_1g_ intensity—a proxy for growth yield—is highest on WS_2_/SiO_2_, intermediate on WS_2_/c-sapphire, and nearly negligible on WS_2_/Si. When this comparison is extended across the WS_2_ thickness series, all three systems show suppression of MoS_2_ on multilayer WS_2_ (*n* ≥ 4). However, in the atomically thin limit (*n* = 0–1), the growth yield remains strongly substrate dependent and is nearly suppressed on Si (Fig. 3d).

The observed growth hierarchy, WS_2_/SiO_2_ > WS_2_/c-sapphire ≫ WS_2_/Si, is fundamentally rooted in the intrinsic electrostatic landscape and surface potential of the underlying substrates. Amorphous SiO_2_ presents a polar oxide surface characterized by a high density of silanol (Si–OH) groups and occasional under-coordinated defects. For bare SiO_2_ (*n* = 0), these surface hydroxyls act as potent chemical anchors: they facilitate the chemisorption of metal–organic precursors through specific surface reactions (e.g., proton transfer or ligand exchange), effectively locking the adatoms in place via strong covalent Mo–O–Si bonds [48–50]. This site-specific chemical trapping significantly lowers the nucleation energy barrier but simultaneously restricts adatom diffusion, leading to the rapid, random "self-seeding" of polycrystalline domains observed on the oxide [51].

In marked contrast, the ionic c-sapphire surface lacks these random chemical traps. Instead, it presents a periodic electrostatic potential that facilitates longer adatom diffusion lengths across a relatively smooth energy landscape. This interpretation is supported by molecular dynamics simulations [52], which demonstrate that crystalline substrates like sapphire exhibit lower energy barriers for adatom migration compared to amorphous oxides. This promotes migration to energetically favorable sites rather than dense stochastic nucleation, resulting in an intermediate growth yield.

Finally, the crystalline Si surface exhibits negligible growth, a behavior attributed to its non-polar, covalent nature. Unlike ionic or hydroxylated surfaces, the neutral Si lattice lacks strong electrostatic dipoles or polar moieties required to anchor the initial precursor molecules [48]. Consequently, the surface exhibits a low adsorption energy, causing reactant molecules to desorb before they can nucleate. We note that Si is typically covered by a thin native oxide; however, its much lower density of hydroxyl terminations and weaker interfacial dipoles compared to thermally grown SiO_2_​ provide insufficient chemical/electrostatic anchoring to stabilize nuclei under our high-temperature MOCVD conditions. This confirms that the substrate’s active (polar/chemical) versus passive (non-polar/inert) character largely dictates the nucleation probability.

Crucially, the preservation of this hierarchy even beneath a WS_2_ interlayer indicates that substrate-specific potentials are not neutralized but are instead transmitted through the atomically thin 2D sheet. For the WS_2_-covered substrates (*n* = 1), direct chemical bonding to the underlying substrate is blocked by the inert vdW surface of WS_2_; instead, the mechanism involves remote electrostatic modulation. Here, substrate-dependent fields (e.g., from polar hydroxyls and defects on SiO_2_) partially penetrate the thin interlayer and locally increase the adsorption energy of precursors on the TMDC surface. These results suggest that an atomically thin WS_2_ layer acts as an electrostatic window, allowing substrate-polarity and defect-related fields to 'leak' through and locally modulate the growth landscape on the chemically inert TMDC surface. As the interlayer becomes thicker than a few layers (*n* ≥ 4), this substrate influence is largely suppressed by the cumulative screening of the TMDC layers, consistent with the thickness-selective overgrowth behavior described in Sect. 3.1.

As discussed below (Sect. 3.3), atomic-resolution STEM further confirms that the TMDC template remains structurally coherent and continuous in growth-active regions, supporting a mechanism dominated by remote modulation of adsorption/chemisorption barriers rather than template defect creation. This substrate-mediated coupling is reminiscent of remote epitaxy, in which the electrostatic potential of a polar or ionic substrate penetrates an ultrathin 2D spacer and can still affect the growth of the overgrown film [36].

### Atomic-scale registry and remote–vdW hybrid epitaxy in MoS_2_/WS_2_

**Fig. 4:**
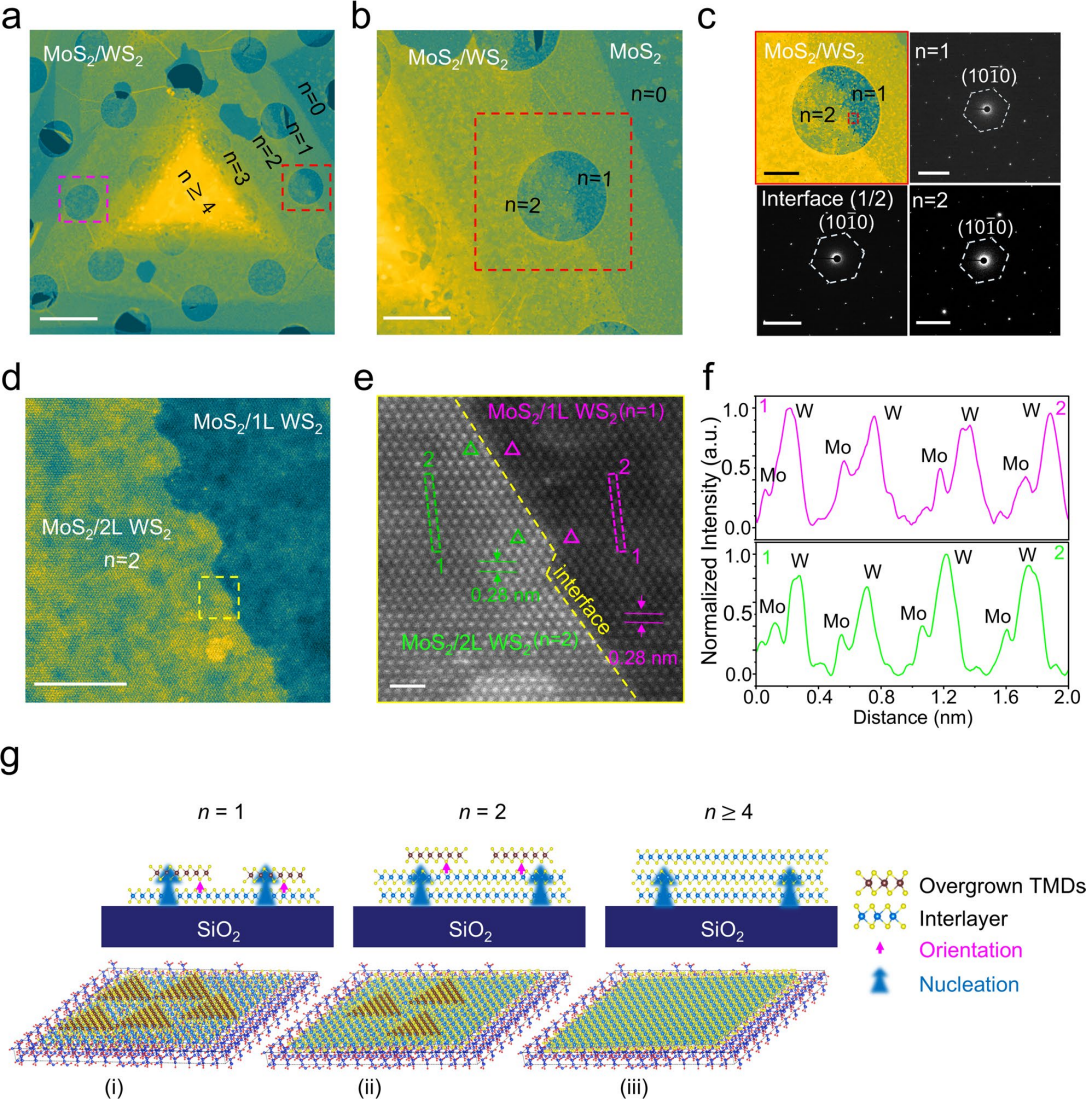
Verification of vdW-dictated registry and the hybrid growth model. **a**, HAADF-STEM survey of a large-area triangular MoS_2_ flake overgrown across a thickness-modulated WS_2_ template, spanning from monolayer (*n* = 1) to multilayer (*n* ≥ 4) regions (scale bar: 2 µm). **b**, Magnified HAADF-STEM view of the interface region (scale bar: 1 µm). **c**, SAED patterns acquired from the *n* = 1, *n* = 2, and interfacial regions, confirming a single set of sharp (10 $\overline{1 }$ 0) hexagonal reflections and invariant crystallographic orientation across thickness boundaries (scale bars: 500 nm; diffraction spot scale: 5 nm.^−1^). **d**, Magnified HAADF-STEM view of the interface region (corresponding to the red dashed box in **c**) showing the boundary between MoS_2_/1L WS_2_ (*n* = 1) and MoS_2_/2L WS_2_ (*n* = 2) regions (scale bar: 10 nm). **e**, Atomic-resolution HAADF-STEM image of the interfacial area (corresponding to the yellow dashed box in **d**), revealing the atomically sharp boundary between MoS₂/1L WS_2_ and MoS_2_/2L WS_2_ with preserved lattice registry (scale bar: 2 nm). **f**, Atomic intensity line profiles corresponding to the dash lines in **e**. The profiles for MoS_2_ on 1L WS_2_ (magenta) and 2L WS_2_ (green) show continuous, periodic atomic-column contrast across the boundary, consistent with an intact stacked heterostructure. **g**, Schematic of the remote–vdW hybrid epitaxy model, illustrating how the 2D template determines the crystallographic orientation of the overgrown layer while the underlying substrate field modulates the nucleation density across varying template thicknesses (*n* = 1, 2, ≥ 4)

Having established that the substrate influences where MoS_2_ can grow through an atomically thin WS_2_ interlayer (Sects. 3.1 and 3.2), we next examine how the WS_2_ itself controls the in-plane crystallographic alignment of the overgrown MoS_2_. To provide a comprehensive assessment, we first performed a large-area HAADF-STEM survey of a triangular MoS_2_ overgrown across a thickness-modulated WS_2_ template, spanning monolayer (*n* = 1) to multilayer (*n* $\ge$ 4) regions (Fig. 4a). The preservation of a single crystallographic orientation across these thickness steps is initially confirmed by reciprocal-space analysis; SAED patterns acquired from the *n* = 1, *n* = 2, and interfacial regions all exhibit a single set of sharp hexagonal (10 $\overline{1 }$ 0) reflections with no detectable spot splitting or rotation (Fig. 4c).

To probe the atomic-scale registry, we examined a representative region where MoS_2_ grows across the 1L–2L WS_2_ boundary (red box in Fig. 4a). Atomically resolved HAADF-STEM images (Fig. 4d, e) show that the in-plane lattice orientation is preserved across the thickness transition, with no contrast reversal or phase shift characteristic of a 60° mirror-twin boundary. The atomic lattice remains continuous across the boundary, with a comparable in-plane spacing (~ 0.28 nm) on both sides, indicating that MoS_2_ maintains a single in-plane registry (i.e., a single-domain registry) across the 1L–2L WS_2_ transition.

Crucially, the registry between the layers is quantitatively verified by atomic intensity line profiles extracted from the *n* = 1 and *n* = 2 regions (Fig. 4f). The profiles for MoS_2_ on 1L WS_2_ (magenta) and 2L WS_2_ (green), resolve the distinct Mo, W, and S columns with high precision. The atomic-column assignment (Mo/W/S) and the stacking-order interpretation for Fig. 4f were validated by multislice HAADF-STEM simulations (Dr. Probe) under matching imaging conditions (Supplementary Note 6; Figs. S11–S12). The periodic intensity peaks confirm that the Mo atomic columns of the overlayer precisely follow the periodic arrangement of the underlying tungsten (W) atoms of the template in both regions.

Collectively, these real- and reciprocal-space observations demonstrate that MoS_2_ forms a single-orientation film that remains crystallographically aligned to the WS_2_ template over the thickness step, consistent with a vdW epitaxial relationship between the two TMDC layers. Notably, these atomic-resolution STEM observations also show that the WS_2_ template remains structurally coherent and continuous in the growth-active regions, with no indication of template degradation that would be expected for defect-mediated nucleation pathways. This supports an interpretation in which the substrate primarily biases the adsorption/chemisorption energy landscape (and thus the sticking coefficient and residence time of adatoms) on a structurally intact 2D surface, rather than generating new template defects as nucleation centers.

Building on this vdW epitaxial alignment and on the thickness- and substrate-dependent growth behavior in Figs. 1–3, we describe our system within a substrate-mediated remote–vdW hybrid picture (Fig. 4g). In classic remote epitaxy, the overlayer can inherit lattice registry directly from the buried substrate because the substrate’s electrostatic potential penetrates an ultrathin 2D spacer [35, 36]. In our fully 2D–2D stacks, by contrast, the in-plane orientation is set by vdW epitaxy to the crystalline WS_2_ interlayer, while substrate-mediated fields primarily modulate adatom adsorption and the subsequent probability and density of nucleation—thereby determining where vertical growth is enabled (for thin interlayer) or suppressed (for thick interlayer). In this sense, our observations extend remote-epitaxy concepts to a “remote–vdW hybrid” regime in which the 2D interlayer fixes the epitaxial registry, whereas the buried substrate tunes the local growth propensity.

Figure 4g schematically summarizes this remote–vdW hybrid picture. Atomically thin WS_2_ interlayers allow substrate fields to bias adatom adsorption and nucleation, enabling vertical overgrowth for *n* ≈ 1–2 (Fig. 4g (i,ii)). In contrast, thicker WS_2_ flakes effectively screen the substrate, so nucleation is strongly suppressed and overgrowth is shut off (Fig. 4g (iii)), consistent with the thickness- and substrate-dependent trends in Figs. 1–3.

### Reciprocal WS_2_/MoS_2_ growth: generality of remote–vdW hybrid epitaxy

**Fig. 5:**
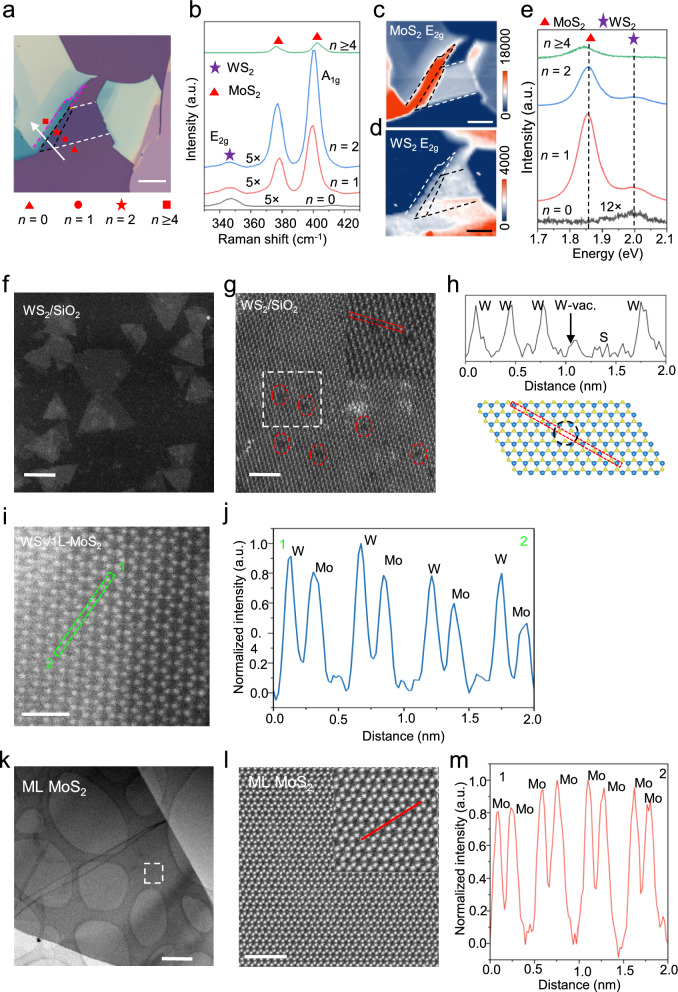
Reciprocal growth behavior and atomic-scale microstructure of WS_2_/MoS_2_ heterostructures. **a**, Optical micrograph of a thickness-graded MoS_2_ template used for reciprocal growth. **b**, Raman spectra showing WS_2_ nucleation on bare SiO_2_ (*n* = 0) and thin MoS_2_ (*n* = 1–2), with complete suppression on multilayer MoS_2_ (*n* ≥ 4). **c**, **d**, Raman intensity maps of the MoS_2_ E_2g_ and WS_2_ E_2g_ modes, confirming the thickness-selective growth window. **e**, Photoluminescence (PL) spectra showing significant emission enhancement for WS_2_ on monolayer (*n* = 1) and bilayer (*n* = 2) MoS_2_ compared to SiO_2_, and the complete suppression of emission on thick MoS_2_ (*n* =  ≥ 4). **f**–**h**, Atomic-resolution STEM images of WS_2_ grown on amorphous SiO_2_, revealing polycrystalline triangular islands with abundant point defects. **i**, **j**, Atomic-resolution STEM of WS_2_ grown on monolayer MoS_2_, showing a continuous lattice with uniform contrast and single in-plane orientation, indicative of coherent heteroepitaxy. **k**–**m**, Atomic-resolution STEM of the multilayer MoS_2_ region resolving only the MoS_2_ lattice, verifying the effective shut-off of WS_2_ nucleation on thick templates. Scale bars: 10 μm (a, c, d); 500 nm (k); 50 nm (f); 2 nm (g, l); 1 nm (i)

To test whether the remote–vdW hybrid mechanism is specific to MoS_2_/WS_2_ or extends to the reciprocal stacking order, we inverted the configuration and investigated WS_2_ growth on MoS_2_ templates (Fig. 5a). Au-assisted exfoliated MoS_2_ flakes on SiO_2_/Si span thicknesses from monolayer (*n* = 1) to multilayer (*n* ≥ 4). Prior to growth, we confirmed these layer assignments by AFM, which yielded a step height of ~ 0.9 nm for the monolayer regions (Supplementary Note 4.1). This precise thickness calibration was then used to label the local layer number *n* in the optical and Raman maps, allowing us to directly test whether the same thickness-dependent overgrowth window appears in the inverted system.

Consistent with the MoS_2_/WS_2_ case, WS_2_ growth occurs preferentially on bare SiO_2_ and atomically thin MoS_2_ (*n* = 1–2) and is strongly suppressed on thicker flakes (Fig. 5b–d). Detailed kinetic analysis and substrate-variant experiments (Si, SiO_2_, c-sapphire) confirm that this selective, thin layer-confined growth is robust to stacking order and primarily driven by the underlying substrate field (Supplementary Notes 4.2–4.3). This thickness-dependent behavior is further corroborated by PL spectra at corresponding regions (Fig. 5e). The WS_2_ emission intensity is significantly higher when grown on monolayer and bilayer MoS_2_ templates compared to that grown on the bare SiO_2_ substrate, while no WS_2_ PL is detected on multilayer MoS_2_ (*n* ≥ 4). This enhancement on thin templates, consistent with our observations in Sect. 3.1, indicates superior crystalline quality and reduced non-radiative recombination in TMDC films supported by vdW interlayers.

Plan-view TEM and SAED provide complementary, larger-area evidence for this trend, showing well-aligned heteroepitaxial WS_2_ on thin MoS_2_, in contrast to polycrystalline WS_2_ domains on SiO_2_ (Supplementary Note 5.1). Within the active thickness window, the MoS_2_ template again dictates the microstructure of the overgrown WS_2_. On amorphous SiO_2_, WS_2_ forms polycrystalline triangular islands characterized by a high density of atomic defects. Atomic-resolution STEM images (Fig. 5f–h) reveal numerous point defects, identified as Tungsten vacancies (highlighted by red circles). The corresponding line intensity profile (Fig. 5h, inset) explicitly shows a sharp drop in atomic column intensity at these sites based on Z-contrast, confirming the absence of W atoms. Additionally, the film can locally develop multilayer patches (2L/3L/ML) during growth (Supplementary Note 5.2).

Atomic-resolution images from these regions exhibit pronounced moiré contrast within the thicker patches (Fig. S9c,e), and the corresponding FFT contains two hexagonal spot sets rotated by ~ 20°, indicating twisted WS_2_ homo-stacking rather than a single, registry-matched multilayer (Fig. S9g, left panel). The extracted moiré periodicity in the filtered image (Fig. S9g, right panel) is consistent with this twist-derived superlattice, highlighting the rotationally unconstrained stacking that is typical when WS_2_ nucleates and thickens on an amorphous oxide surface. The high density of grain boundaries and surface-induced disorder in these polycrystalline domains likely serve as potent non-radiative quenching centers, explaining the weak PL emission observed on SiO_2_.

In marked contrast, WS_2_ grown on monolayer MoS_2_ forms a continuous lattice with highly uniform Mo/W/S column contrast. Line intensity profiles across these regions show consistent atomic intensities with no evidence of the vacancy-induced dips observed on SiO_2_ (Fig. 5i,j; Fig. S13). Furthermore, the corresponding FFT confirms a single in-plane orientation indicative of coherent heteroepitaxy. This distinct difference demonstrates that the monolayer template not only aligns the crystal orientation but also strongly suppresses vacancy formation compared to growth on amorphous substrates. This transition from polycrystalline growth on SiO_2_ to single-orientation vdW epitaxy on the MoS_2_ template effectively minimizes non-radiative decay channels. By providing an atomically smooth, dangling-bond-free surface that screens the overlayer from the underlying substrate’s disordered electrostatic landscape, the thin MoS_2_ template facilitates the growth of TMDC layers with markedly improved optical quality.

In regions where the underlying MoS_2_ exceeds several layers, only the MoS_2_ lattice is resolved, with no additional periodic contrast attributable to WS_2_ (Fig. 5j–l), confirming that WS_2_ nucleation is effectively shut off on thick templates. Cross-sectional STEM and EDS analysis further verify this "shut-off" mechanism: while active regions show a distinct, chemically sharp WS_2_/MoS_2_ hetero-stack, the thick-template regions reveal a multilayer MoS_2_ surface with no overgrowth (Supplementary Note 5.3). We note that the cross-sectional images also show an atomically continuous TMDC template with no discernible pinholes or open channels within the inspected regions. This structural continuity stands in marked contrast to pinhole- or 'thru-hole'-assisted epitaxy reported in systems with discontinuous spacers [53–55].

Our observations therefore argue against defect-mediated growth pathways, suggesting instead that nucleation is modulated by field penetration through the atomically continuous lattice. These reciprocal-growth experiments show that the remote–vdW hybrid regime is robust to stacking order: in both MoS_2_/WS_2_ and WS_2_/MoS_2_, an atomically thin TMDC interlayer sets the in-plane registry wherever growth occurs, while the buried substrate and interlayer thickness jointly control whether nucleation is enabled or suppressed. These findings establish remote–vdW hybrid epitaxy as a general design principle for 2D–2D vertical TMDC heterostructures.

## Conclusions

In summary, we have demonstrated a substrate-field-modulated remote–vdW hybrid epitaxy regime, where the in-plane registry is dictated by vdW coupling to the 2D template. This approach yields single-orientation heterostructures with no detectable mirror-twin signatures within the examined areas for both reciprocal stacking orders. This dual-control mechanism provides a robust strategy for the deterministic synthesis of high-quality 2D semiconductor heterostructures, addressing key limitations of conventional epitaxial growth. Beyond its role in growth control, the resulting interfacial charge environment may influence the electrical performance of these heterostructures. Potential effects include substrate-induced doping, modified charge-transfer kinetics, and gate-dependent hysteresis, offering a new degree of freedom for substrate-engineered device tuning. Our work establishes a practical framework for the large-area, scalable synthesis of high-quality vertical heterostructures, providing a versatile pathway for developing next-generation electronic and optoelectronic devices with precisely engineered 2D interfaces.

## Supplementary Information

Additional file1 (DOCX 7068 kb)

## Data Availability

The data supporting the findings of this study are available within this article and its Supplementary Information. Source data are provided with this paper.

## Abbreviations

2D: Two-dimensional
AFM: Atomic force microscopy
CVD: Chemical vapor deposition
DES: Diethyl sulfide
EDS: Energy-dispersive X-ray spectroscopy
FFT: Fast Fourier transform
FIB: Focused ion beam
HAADF-STEM: High-angle annular dark-field scanning transmission electron microscopy
HR-TEM: High-resolution transmission electron microscopy
HS: Heterostructure
ML: Multilayer
MOCVD: Metal–organic chemical vapor deposition
OM: Optical microscopy
PL: Photoluminescence
SAED: Selected-area electron diffraction
STEM: Scanning transmission electron microscopy
TMDC: Transition-metal dichalcogenide
vdW: Van der Waals
WHC: Tungsten hexacarbonyl
