# Supplementary material for: Substrate-field-modulated remote-van der Waals hybrid epitaxy in transition metal dichalcogenide heterostructures
Source: Nano Converg. 2026 Mar 28;13:12. doi: 10.1186/s40580-026-00542-4 (PMC13033003; doi:10.1186/s40580-026-00542-4)
Supplement: Supplementary file 1 — Additional file1 (DOCX 7068 kb) [file 40580_2026_542_MOESM1_ESM.docx]

Supplementary Information for Substrate-field-modulated remote-van der Waals hybrid epitaxy in transition metal dichalcogenide heterostructures

Lia Saptini Handriani^1^, Suhee Jang^1^, Yelim Kim^1^, Hyuncheol Yun^1^, Dae Yeop Jeong^1^, Hyeonsu Park^1^, Zhe Gao^1^, Jae-il Jang^1^, and Won Il Park^1^*

^1^Division of Materials Science and Engineering, Hanyang University, Seoul, 04763, Republic of Korea.

*Corresponding author: Won Il Park

Tel.: +82-2-2220-0504

E-mail address: [wipark@hanyang.ac.kr](mailto:wipark@hanyang.ac.kr)

**CONTENTS**

Supplementary Note 1. Multi-Gaussian Peak Fitting of Raman and PL Mapping Data 3

Supplementary Note 2. AFM Analysis of Substrate-Dependent MoS_2_ Accumulation 6

Supplementary Note 3. Order-of-magnitude estimate of Debye screening length 7

Supplementary Note 4. Reciprocal WS_2_/MoS_2_ growth: thickness window, growth kinetics, and
substrate dependence 8

Supplementary Note 5. Atomic-Scale Structural and Interface Analysis 12

Supplementary Note 6. Multislice HAADF-STEM Simulations 20

**Supplementary Note 1. Multi-Gaussian Peak Fitting of Raman and PL Mapping Data**

To accurately quantify the overgrowth behavior and spatial distribution of the heterostructures, we employed a multi-Gaussian peak fitting procedure rather than simple peak-intensity integration. This approach is essential for deconvoluting overlapping spectral features and removing background artifacts, as illustrated in **Figure S1-2**.

**Rationale and Methodology**

In vertical MoS_2_/WS_2_ heterostructures, the phonon modes of the two materials often lie in close proximity. Specifically, the MoS_2_ A_1g_ mode (~401 cm^-1^) and the WS_2_ A_1g_ (~414 cm^-1^) appear in the same spectral window. In standard “raw” intensity mapping (which simply selects the maximum intensity within a specific range), the tail of a strong signal from the underlying template can bleed into the detection window of the weaker overlayer peak, leading to artificial contrast and overestimation of its intensity.

To address this, we processed the Raman mapping datasets using the following steps:

1. **Baseline Subtraction:** A polynomial baseline was subtracted from each raw spectrum to eliminate background fluorescence or scattering variations caused by the substrate.
2. **Multi-Gaussian Fitting:** The spectra were fitted using multiple Gaussian functions to deconvolute individual peak contributions. This allows for the precise separation of the MoS_2_ A_1g_ intensity from the adjacent WS_2_ A_1g_ peak.
3. **Handling of Baseline Noise (Non-negative Constraint):** In regions where MoS_2_ growth is negligible or absent (e.g., on thick WS_2_ layers), the Raman signal intensity is effectively zero. In these signal-void regions, subtracting the baseline from the raw spectra can occasionally result in small, mathematically negative values due to random spectral noise fluctuations. Since negative intensity has no physical meaning, these numerical artifacts were set to zero (clamped) in the quantitative plots (e.g., Fig. 2e and Fig. 3d) to accurately represent the absence of material and avoid ambiguity.

The mapping parameters were optimized for each sample set: the MoS_2_/WS_2_ maps (Fig. S1a–e) comprise 2,601 spectra collected across a 51 × 51 grid, while the reciprocal WS_2_/MoS_2_ maps (Fig. S2a–e) consist of 3,721 spectra collected across a 61 × 61 grid.

A similar multi-peak Gaussian fitting protocol was applied to the photoluminescence (PL) mapping datasets. This ensured that exciton peak intensities and positions (e.g., in Figure 1g, h) were extracted accurately, independent of variations in background level or spectral broadening.

**Analysis of MoS_2_/WS_2_ (Fig. S1a–e)**

Fig. S1b shows a representative raw spectrum where the MoS_2_ and WS_2_ peaks overlap. Maps generated without fitting (Fig. S1c) often show ambiguous contrast due to this spectral congestion. By applying the fitting algorithm (Fig. S1d), we successfully isolated the individual peak components. The resulting fitted intensity maps (Fig. S1e) reveal a much clearer spatial distribution of the MoS_2_ overlayer, confirming that accurate identification of the active overgrowth regions requires peak deconvolution rather than simple intensity integration.


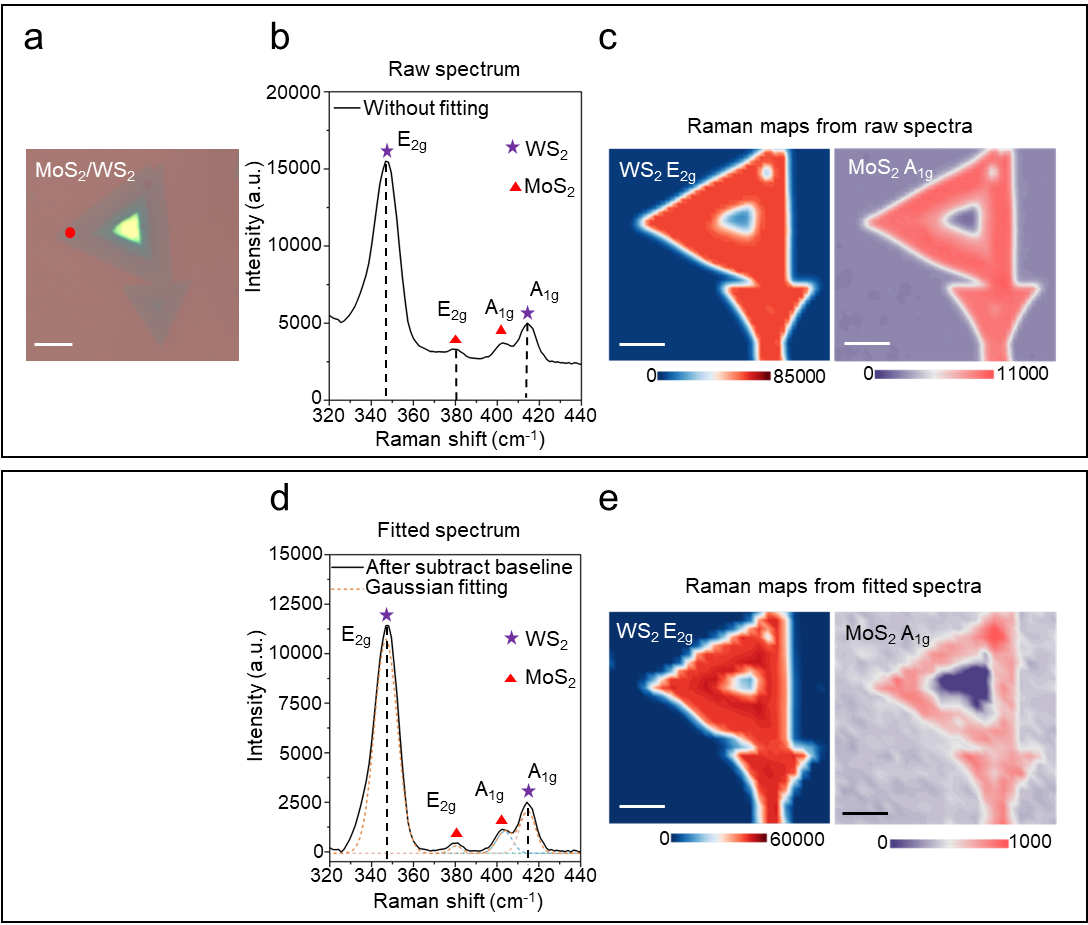


**Fig. S1 | Multi-Gaussian fitting of Raman mapping data for MoS_2_/WS_2_**. **a,** Optical image of MoS_2_ growth on a triangular WS_2_ flake. **b,** Representative raw Raman spectrum from an overlap region, showing the congestion between MoS_2_ and WS_2_ phonon modes. **c,** Raman intensity maps for WS_2_ E_2g_ and MoS_2_ A_1g_ peaks generated by simple integration without fitting, showing ambiguous contrast due to signal overlap. **d,** The spectrum from **b** after baseline subtraction and multi-Gaussian fitting. The individual Gaussian components (dashed lines) illustrate the robust deconvolution of the overlapping MoS_2_ A_1g_ (~401 cm⁻¹) and WS_2_ A_1g_ (~414 cm⁻¹) peaks. **e,** Corresponding Raman intensity maps generated from the fitted peak areas, revealing a much clearer spatial distribution of the MoS_2_ overlayer. Scale bars in panels a–e represent 5 μm.

**Analysis of WS_2_/MoS_2_ (Fig. S2a–e)**

We applied the same rigorous analysis to the inverted WS_2_/MoS_2_ system. Although the stacking order is reversed, the spectral overlap between the vibrational modes remains. Fig. S2b and S2d compare the spectra before and after fitting, respectively. The fitted maps (Fig. S2e) clearly distinguish the WS_2_ E_2g_ and A_1g_ signals from the underlying MoS_2_ template, providing a reliable measure of growth yield on different MoS_2_ thicknesses (monolayer vs. multilayer).

By realistically separating the contributions of individual peaks, this multi-Gaussian analysis significantly improves the reliability of the spectroscopic maps that underpin the thickness-selective growth windows reported in the main text.

**
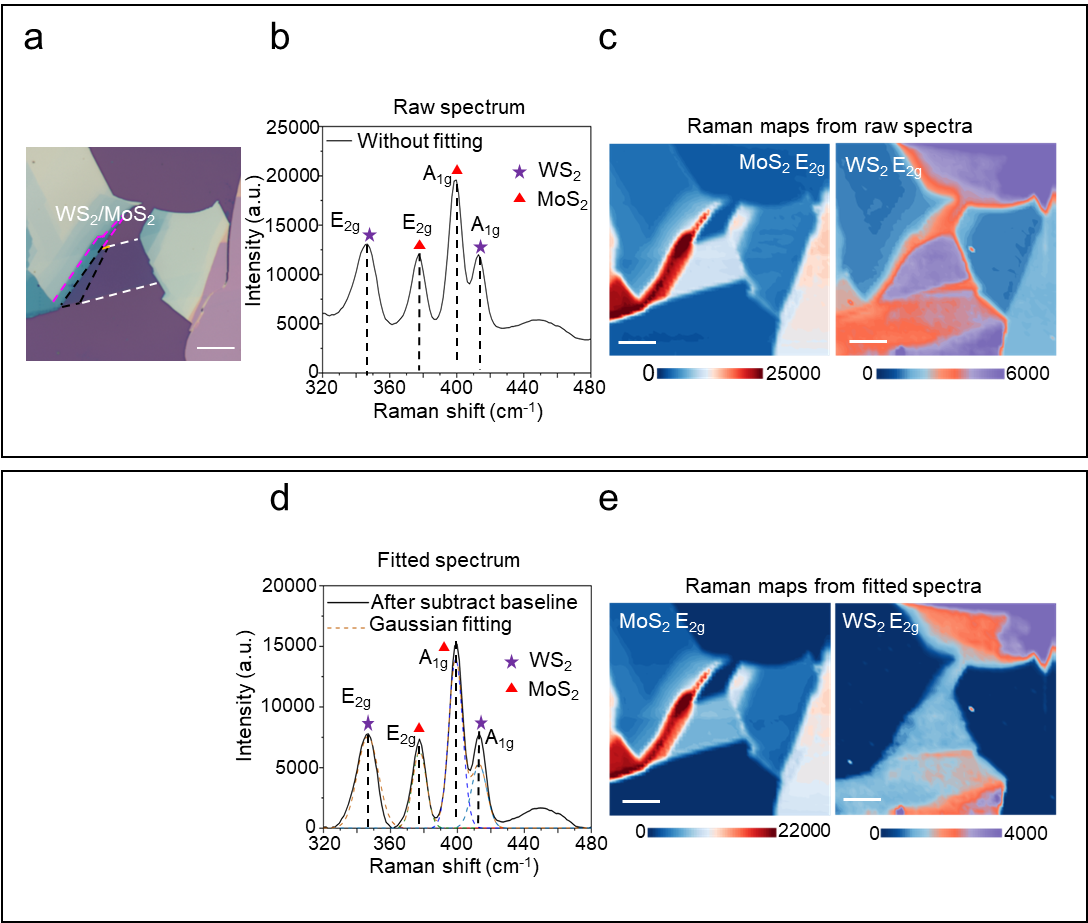
**

**Fig. S2 | Multi-Gaussian fitting of Raman mapping data for WS_2_/MoS_2_ heterostructures**. **a–e,** Analogous analysis for the reciprocal WS_2_/MoS_2_ system. **a,** Optical image of WS_2_ grown on an exfoliated MoS_2_ flake. **b,** Raw Raman spectrum showing peak overlap. **c,** Intensity maps of MoS_2_ E_2g_ and WS_2_ E_2g_ generated without fitting. **d,** The spectrum from **b** after baseline subtraction and multi-Gaussian fitting, resolving the individual MoS_2_ and WS_2_ contributions. **e,** Fitted Raman intensity maps showing improved contrast and reliable separation of the WS_2_ growth from the underlying MoS_2_ template. Scale bars in panels a–e represent 10 μm.

**Supplementary Note 2. AFM Analysis of Substrate-Dependent MoS_2_ Accumulation**

To corroborate the spectroscopic findings and decouple the effects of physical accumulation from potential changes in crystallinity or grain coalescence, we performed AFM characterization of the MoS_2_ overlayers. While Raman and PL mapping provide a sensitive measure of the presence of material, AFM provides a direct, independent measure of vertical film thickness and topographic height.

As shown in Fig. S3, the vertical accumulation of MoS_2_ differs significantly depending on the underlying surface. Line profiles reveal that the physical height of MoS_2_ on the bare SiO_2_ substrate is consistently greater than on the WS_2_ template. This height analysis confirms that the time-dependent Raman intensity rise observed in Fig. 2 reflects a genuine increase in material volume (vertical thickness) rather than solely reflecting lateral expansion of areal coverage or changes in crystallinity.

This observation is consistent with substrate-dependent growth propensity: the amorphous SiO_2_ surface, possessing higher surface energy and abundant dangling bonds, promotes denser nucleation and faster vertical accumulation. In contrast, the chemically inert, van der Waals surface of the WS_2_ template suppresses vertical growth, leading to a thinner, more uniform overlayer. In this way, the AFM data reinforces the Raman and PL results (Fig. 1c–h), confirming that the MoS_2_ overgrowth on WS_2_ is confined to a thin, controlled epilayer, whereas the oxide substrate drives the formation of thicker, uncontrolled deposits.

**
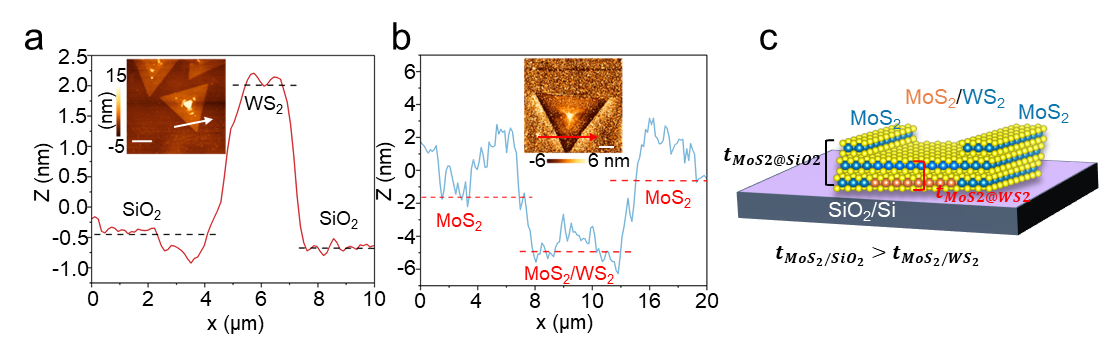
**

**Fig. S3 | AFM characterization of differential MoS_2_ accumulation**. **a,b,** AFM topography images of (a) WS_2_ flakes grown on a SiO_2_ substrate and (b) MoS_2_ overgrowth on WS_2_ flakes and the adjacent bare SiO_2_ region. The scan covers the boundary between the bare SiO_2_ substrate and the WS_2_ domain. The height profile corresponding to the line scan indicated in the inset. The step height analysis reveals a larger physical thickness for the MoS_2_ grown on SiO_2_ compared to that on the WS_2_ surface. This independent height measurement substantiates that the Raman intensity trends in Fig. 2 correspond to actual vertical material accumulation. **c,** Schematic illustration summarizing the growth kinetics: high nucleation density and rapid vertical build-up occur on the reactive SiO_2_ surface, while the inert basal plane of the WS_2_ template promotes slower, surface-limited growth. Scale bars in the insets represent 5 μm.

**Supplementary Note 3. Order-of-magnitude estimate of Debye screening length**

**(i) Debye screening length**

To provide a quantitative basis for the thickness-dependent “active window”, we estimate the Debye screening length ($\text{λ}_{D}$) of mobile carriers in the atomically thin TMDC template at the growth temperature (650 °C) using:

$\text{λ}_{D}=\sqrt{\varepsilon_{0}\varepsilon_{r,\perp}K_{B}T/(e^{2}n)}$ — (S1)

where $\varepsilon_{0}$ is the vacuum permittivity, $\varepsilon_{r,\perp}$ is the out-of-plane relative permittivity, $K_{B}$ is the Boltzmann constant, $e$ is the elementary charge, and $n$ is the effective 3D carrier density.

**(ii) Relative permittivity for MoS_2_ and WS_2_**

Since the substrate field penetrates normal to the layers, we adopt the static out-of-plane permittivity $\varepsilon_{r,\perp}$ ≈ 6.2 for MoS_2_ and 6.1 for WS_2_ [1].

**(iii) Carrier Density for MoS_2_ and WS_2_**

At 650 °C, the carrier density $n$ is significantly enhanced by thermal excitation and unintentional doping from sulfur vacancies. Based on typical sheet densities ($n_{s}$ ≈ 10^12^–10^13^ cm^-2^ and converting to 3D density ($n=n_{s}/t$, where t is the layer thickness), we adopt an effective density range of $n$ ≈ (0.6–6) × 10^19^ cm^-3^.

**(iv) Results and Correlation**

Substituting $T$ = 650 °C (923 K), $\varepsilon_{r,\perp}$ = 6.1–6.2, and $n$ = (0.6–6) × 10^19^ cm^-3^ into Eq. (S1) yields:

$\text{λ}_{D}$ ≈ 0.7–2.2 nm — (S2)

Given a monolayer thickness of ~0.65 nm, this corresponds to $\text{λ}_{D}$ ≈ 1–3.4 layers. This theoretical result is in excellent agreement with the experimentally observed growth window (~1–3 layers), confirming that the substrate-field-modulated nucleation is limited by the electrostatic screening length of the 2D interlayer at the growth temperature.

**Supplementary Note 4. Reciprocal WS_2_/MoS_2_ growth: thickness window, growth kinetics, and substrate dependence**

**4.1. Thickness verification and spatial selectivity**

To strictly correlate the WS_2_ growth behavior with the MoS_2_ template thickness as shown in Fig. 5, an accurate layer-number assignment of the flakes was essential. Prior to WS_2_ deposition, we performed AFM on the exfoliated MoS_2_ templates. As shown in Fig. S4, the topographic step height analysis yields a thickness of ~0.9 nm for the thinnest regions, corresponding to a monolayer (*n* = 1), and successive steps corresponding to integer multiples of this value. This calibration was used to label the optical and Raman maps in Fig. 5a. With this thickness confirmed, the Raman mapping in Fig. 5c,d demonstrates that WS_2_ nucleation is spatially confined to the bare SiO_2_ and the *n* =1–2 MoS_2_ regions. The abrupt disappearance of the WS_2_ signal upon crossing into thicker MoS_2_ (*n* ≥ 4) regions confirms that the suppression mechanism is robust and intrinsic to the template thickness.


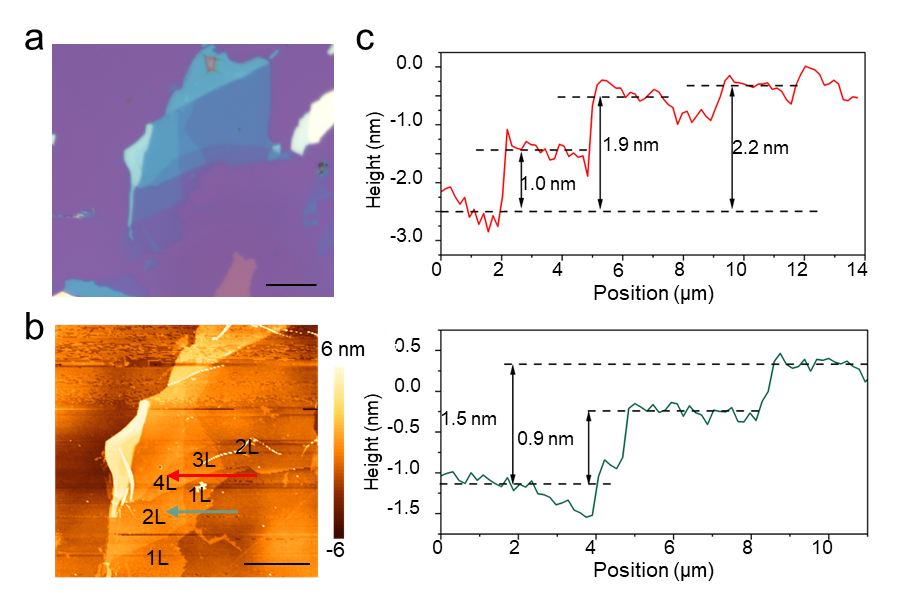


**Fig. S4 | Thickness calibration of exfoliated MoS₂ templates.** **a**, OM image of a representative MoS₂ flake used for layer-number (*n*) assignment. **b**, AFM height map of the same region showing stepped terraces. **c**, AFM line profiles along the colored traces in b, confirming discrete step heights used to calibrate local MoS₂ layer number *n* prior to WS₂ growth. Scale bars in (a) and (b) are 10 µm.

**4.2. Time-resolved growth evolution in WS_2_-on-MoS_2_ heterostructures**

To differentiate between nucleation barriers and vertical growth rates, we tracked the evolution of the WS_2_ signal on the MoS_2_ template over a range of deposition times (7, 10, and 15 min).

- **Nucleation Phase (5–7 min):** The growth kinetics are characterized by a distinct nucleation delay. During the first 5 minutes, negligible signal is detected, reflecting the kinetic barrier required to establish stable nuclei. By 7 minutes, however, WS_2_ Raman signatures emerge clearly on the monolayer MoS_2_ (*n* = 1) regions, whereas the signal on the bare SiO_2_ remains close to the noise floor (Fig. S5a–c, j). This indicates that the crystalline MoS_2_ template lowers the nucleation energy barrier, facilitating earlier adatom capture compared to the amorphous substrate.
- **Vertical Build-up Phase (10–15 min):** With continued deposition, WS_2_ nucleates on the SiO_2_ surface and grows rapidly. As shown in Fig. S5d–i, the rate of signal increase on SiO_2_ significantly exceeds that on the monolayer MoS_2_. This confirms that while the oxide surface has a higher nucleation barrier (longer delay), it promotes faster vertical accumulation once growth begins, likely due to a higher density of active surface sites compared to the van der Waals surface of the template.
- **Persistent Suppression:** Crucially, the multilayer MoS_2_ regions (*n* ≥ 4) show no appreciable WS_2_ signal throughout the entire time series (Fig. S5l). This confirms that the suppression of nucleation on thick templates is stable and is not simply a result of delayed kinetics, but rather a fundamental lack of adatom capture due to the screening of the substrate field.


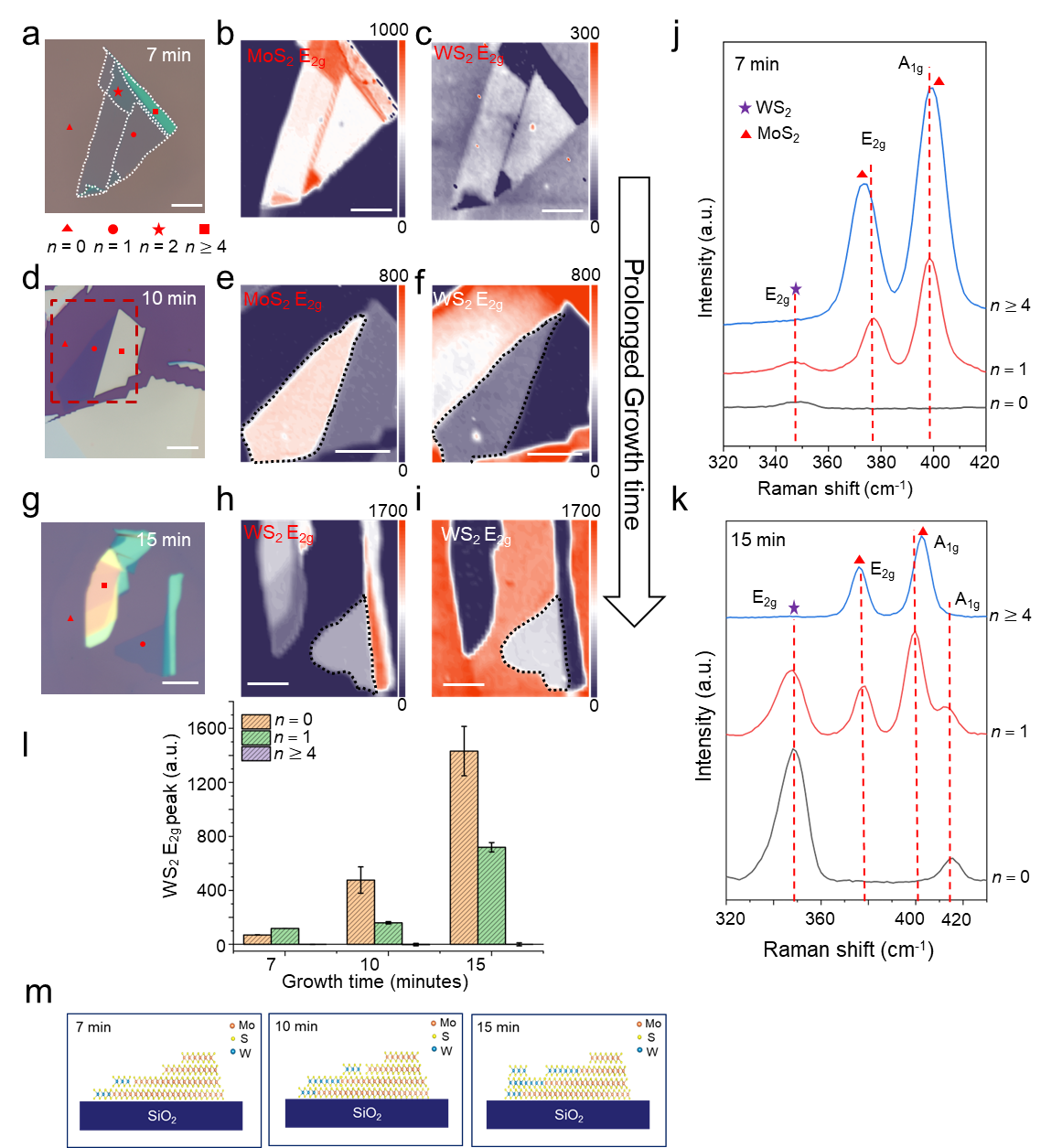


**Fig. S5 | Growth kinetics in the reciprocal WS_2_-on-MoS_2_ configuration. a–c, 7 min growth:** **a**, OM image of the MoS_2_ thickness-gradient flake with markers indicating thickness-defined regions (*n* = 0, 1, 2 and ≥4). **b**, Raman intensity map of the MoS_2_ E_2g_ mode outlining the template terraces. **c**, Corresponding WS_2_ E_2g_ map showing the emergence of WS_2_ signal on bare SiO_2_ and ultrathin MoS_2_ regions**. d–f**, 10 min growth: **d**, OM (boxed region indicates the mapped area), **e**, MoS_2_ E_2g_ map, and **f,** WS_2_ E_2g_ map. **g–i,** 15 min growth: **g,** OM, **h,** MoS_2_ E_2g_ map, and **i,** WS_2_ E_2g_ map. Across all time points, WS_2_ signal remains negligible on multilayer MoS_2_ (*n* ≥ 4). Dashed outlines indicate the MoS_2_ flake boundary. **j,k**, Representative Raman spectra from the marked positions at 7 min **(j)** and 15 min **(k);** vertical dashed lines mark characteristic MoS_2_ and WS_2_ phonon modes. **l**, Quantified WS_2_ E_2g_ peak-area statistics extracted from the thickness-defined regions as a function of growth time (mean ± s.e.m.), showing sustained signal buildup on growth-active regions and minimal response on thick MoS_2_. **m**, Schematic summary of the time evolution, illustrating delayed nucleation followed by preferential vertical build-up on bare and ultrathin-template regions. Scale bars, 20 µm (a–f) and 10 µm (g–i).

**4.3. Substrate-dependent nucleation on MoS_2_**

To verify that the substrate hierarchy observed in the main text (Section 3.2) is not specific to the WS_2_ template but is a general feature of the remote–vdW hybrid mechanism, we analyzed the reciprocal growth of WS_2_ on MoS_2_ templates transferred onto Si, SiO_2_, and c-sapphire.

- **Thickness Calibration:** To ensure a valid comparison across varying optical backgrounds, the MoS_2_ layer number on each substrate was calibrated using the frequency difference (Δω) between the MoS_2_ E_2g_ and A_1g_ Raman modes. As shown in Fig. S6a, this metric provides a consistent, substrate-independent standard for identifying monolayer (*n* =1) to multilayer regions (*n* ≥ 4).
- **Growth Hierarchy:** The statistical analysis of the WS_2_ E_2g_ intensity reveals that the growth yield follows the same order as the direct case: SiO_2_ > c-sapphire ≫ Si.
- **Suppression on Si:** Significantly, on the Si substrate, the WS_2_ signal remains negligible not only on the thick MoS_2_ regions but also on the monolayer MoS_2_ and the bare Si surface itself. These data confirm that the presence of an atomically thin template alone is insufficient to promote nucleation if the underlying substrate does not provide the necessary electrostatic support, reinforcing the dual-control mechanism proposed in the main text.


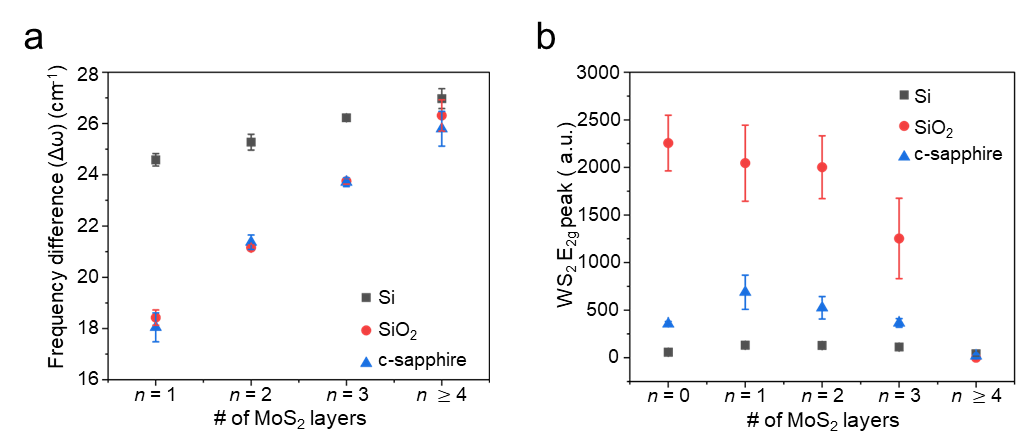


**Fig. S6 |** **Substrate-dependent growth of WS_2_ on MoS_2_ templates.** **a,** Calibration of the MoS_2_ template thickness on different substrates (Si, SiO_2_, and c-sapphire). The layer number (*n*) is assigned based on the frequency difference (Δω) between the MoS_2_ E_2g_ and A_1g_ Raman modes, ensuring consistent identification of monolayer (*n* = 1) to multilayer regions across all support materials. **b,** Statistical comparison of the WS_2_ growth yield, represented by the WS_2_ E_2g_ peak intensity, as a function of MoS_2_ template thickness on the three substrates. The data confirms a hierarchy of growth efficiency (SiO_2_ > c-sapphire ≫ Si) and demonstrates that nucleation is suppressed on thick MoS_2_ templates regardless of the substrate, while the yield on thin MoS_2_ is strongly modulated by the underlying support.

**Supplementary Note 5. Atomic-Scale Structural and Interface Analysis**

**5.1. Broad-area survey of crystallographic alignment**

The structural integrity of the synthesized heterostructures is rooted in the initial template-directed growth sequence. To verify this, we performed multi-scale structural analyses for both stacking configurations.

First, for the primary MoS_2_ on WS_2_ (forward) growth, multi-location HAADF-STEM and FFT analyses were conducted to assess the lattice registry across template thickness boundaries (*n* = 1–2). Atomic-resolution imaging confirms that crystalline continuity is preserved across these thickness steps (Fig. S7), indicating that the MoS_2_ overlayer establishes a robust, single-crystalline 2D registry that remains undisturbed by local variations in the underlying template thickness. As shown in the HAADF-STEM surveys and corresponding FFTs (Fig. S7a, b), a single set of sharp hexagonal reflections is consistently preserved across the boundary regions within the examined fields of view. High-resolution images (Fig. S7c–f) further resolve an atomically sharp and continuous lattice, supporting the conclusion that the in-plane registry is dictated by the 2D template rather than the amorphous SiO_2_ support.

To further demonstrate the macroscopic reproducibility of this template-locked alignment, we performed a large-area survey on the reciprocal WS_2_ on MoS_2_ (reverse) sequence (Fig. S8). Plan-view TEM and SAED were employed to compare growth on bare SiO_2_ versus the MoS_2_ template. SAED acquired from the bare SiO_2_ regions (*n* = 0) exhibits a ring-like/broadened diffraction signature, consistent with multiple in-plane orientations in the absence of a crystalline template (Fig. S8b, h). In contrast, SAED patterns from MoS_2_-supported regions (*n* = 1–2) consistently show sharp, discrete hexagonal diffraction spots (Fig. S8c, d), indicating a single in-plane registry set by the template. This orientation consistency is further validated by a systematic survey across multiple flakes and distant regions on the substrate (Fig. S8e–l), where identical single-registry SAED signatures are observed.

Collectively, these multi-scale observations across both growth sequences—from atomic-scale lattice continuity to macroscopic SAED surveys—provide strong evidence for a template-locked registry model. This mechanism ensures highly preferred orientation on amorphous SiO_2_ supports, where no crystalline substrate lattice imprinting is possible.


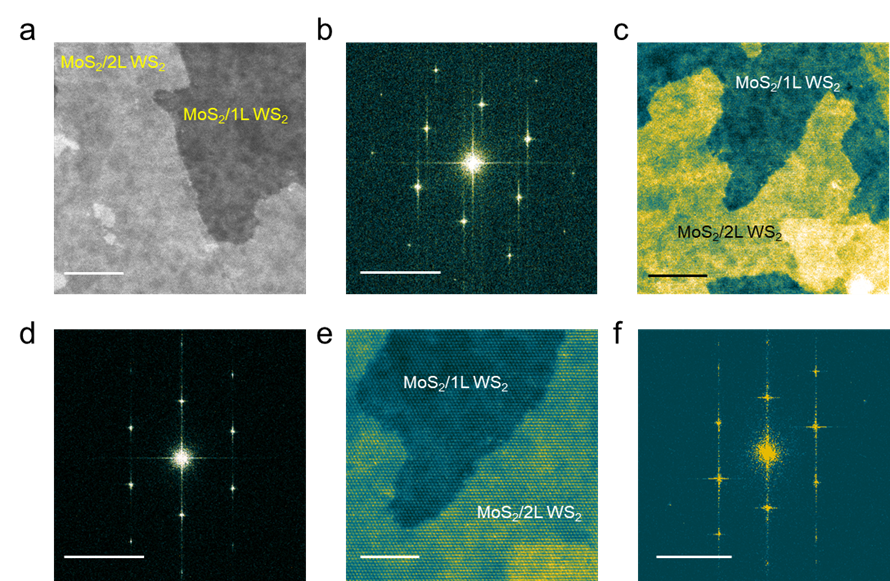


**Fig. S7 | Multi-location HAADF-STEM and FFT analysis across thickness boundaries. a–b**, HAADF-STEM survey and corresponding Fast Fourier Transform (FFT) of the red-box region (from Figure 4a) showing the MoS_2_/1L-WS_2_ (*n* = 1) to MoS_2_/2L-WS_2_ *(n* = 2) boundary. The FFT displays a single hexagonal reflection set, confirming epitaxial alignment across the thickness step. **c–f**, HAADF-STEM images and FFTs from the magenta-box region (from Figure 4a). Atomic-resolution imaging (e) resolves the sharp interface between MoS_2_/1L-WS_2_ and MoS_2_/2L-WS_2_, with the FFT (f) confirming crystalline continuity. Scale bars: 10 nm (a,c); 5 nm (e). Reciprocal-space scale bars: 5 nm^-1^ (b,d,f).

**
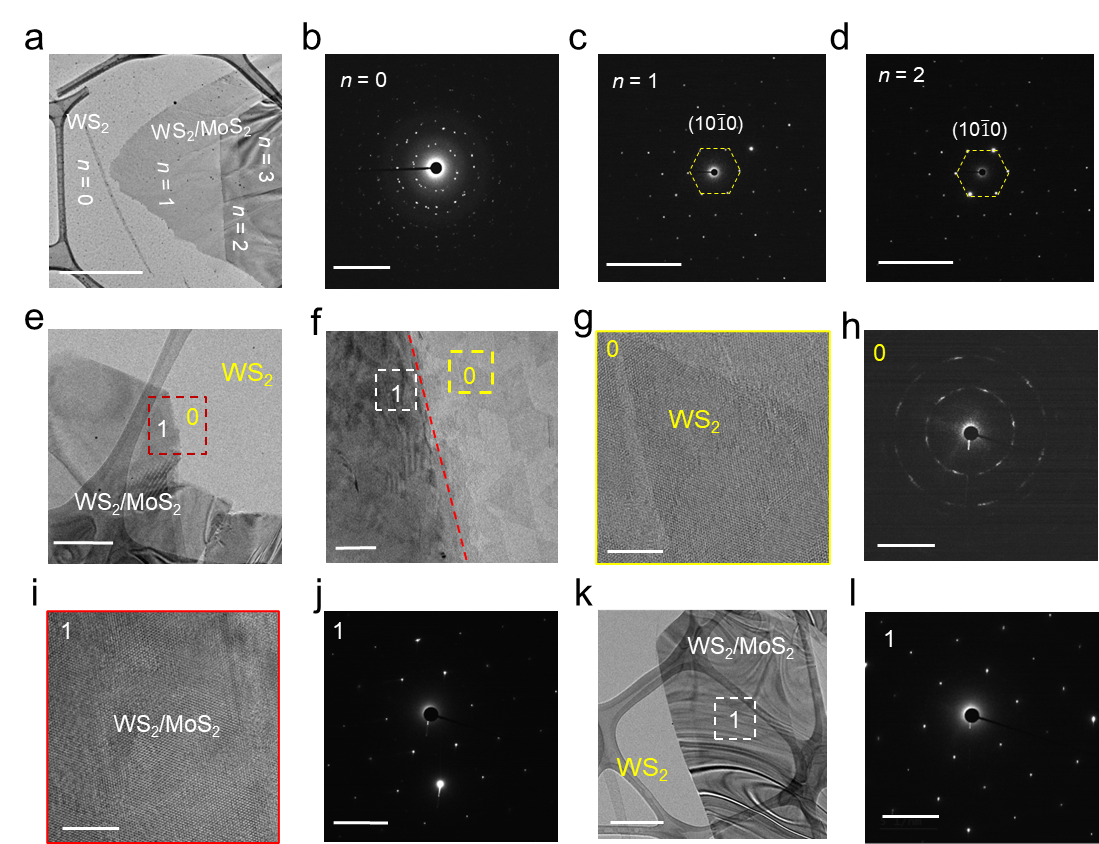
**

**Fig. S8 | Broad-area orientation survey of template-directed WS_2_ growth on mechanically exfoliated MoS_2_ templates. a,** Plan-view TEM image of a representative thickness-gradient region with the local MoS_2_ layer number (*n*) labeled (*n* = 0–3). **b–d,** SAED patterns acquired from the regions indicated in **a**: **b,** *n* = 0 (bare SiO_2_), **c,** *n* = 1, and **d,** *n* = 2. The bare region exhibits a ring-like/broadened diffraction signature, whereas thin MoS_2_ regions show discrete hexagonal spots, indicating template-locked in-plane registry. **e**, Low-magnification plan-view TEM survey covering multiple representative WS_2_/MoS_2_ flakes demonstrating the macroscopic reproducibility of the growth behavior across the sample. **f,** Low-magnification TEM image showing the coexistence of Region 0 (bare SiO_2_) and Region **1** (MoS_2_-templated surface) within a single flake area. **g, i,** High-magnification plan-view TEM images of the areas boxed in **f**, corresponding to WS_2_ growth on SiO_2_ (**g**) and on the MoS_2_ template (**i**). **h,j,** Corresponding SAED patterns acquired from the same areas as g and i, respectively; h shows a polycrystalline ring-like signature in the absence of a crystalline 2D template, whereas j exhibits sharp hexagonal (10$\bar{1}0$) reflections, confirming a well-defined in-plane orientation locked to the underlying MoS_2_ template. k, Additional SAED acquired from a different position on the sample, showing consistent hexagonal (10$\bar{1}0$) reflections and thus confirming orientation reproducibility across surveyed regions. Scale bars :1 μm in panels **a**; 10 nm^-1^ in **b**–**d**; 200 nm in **e**; 20 nm in **f**, 5 nm in g and i, and 5 nm^-1^ in **h, j, l**, 500 nm in **k**.

**5.2 Analysis workflow for stacking/registry identification**

To support the microstructure discussion in Fig. 5f–m, Fig. S9 provides representative atomic-resolution images acquired from regions corresponding to WS_2_ grown on bare SiO_2_, monolayer MoS_2_, and multilayer MoS_2_ within the same reciprocal-growth experiment. Here we summarize the analysis procedure and the criteria used to distinguish (i) rotationally misaligned WS_2_ multilayer patches on SiO_2_, (ii) single-orientation WS_2_ on monolayer and few layer MoS_2_, and (iii) absence of WS_2_ on thick templates.

**(i) Identification of twisted WS_2_ multilayer patches on SiO_2_**
For the locally thickened WS_2_ patches observed on SiO_2_ (Fig. S9b–e), we performed FFT analysis on the atomic-resolution images. The presence of two hexagonal spot sets —shown in the left panels of Fig. S9f (for region c) and Fig. S9g (for region e)—serves as the primary indicator of relative rotational misalignment and twisted homo-stacking. To visualize the resulting superlattice modulation, a band-pass/spot-filtering procedure was applied in the frequency domain to isolate the moiré component. The reconstructed periodic contrast is visualized in the right panel of Fig. S9f and Fig. S9g, enabling the extraction of the moiré periodicity (~0.84–0.86 nm at 61°). This quantitative descriptor of the twist-induced superlattice confirms the rotationally unconstrained nature of growth on amorphous supports.

**(ii)** **Verification of single-registry WS_2_ on monolayer MoS_2_ and few layer MoS_2_ ( *n* = 1-3)**
For WS₂ grown on monolayer MoS_2_ templates (Fig. 5i,j and Supplementary S9h-j), the epitaxial registry was assessed by the absence of split/duplicated hexagonal spot sets in the SAED/FFT over the analyzed field of view. A critical finding is the consistent "single diffraction" signature observed across a thickness gradient from *n* = 1 to *n* = 3 (Fig. S9i–l). SAED/FFT patterns acquired from monolayer (*n* = 1), bilayer (*n* = 2), and trilayer (*n* = 3) regions all exhibit sharp, discrete hexagonal spots, confirming that the in-plane orientation remains locked to the underlying template regardless of its local thickness. Spatial uniformity of the atomic lattice contrast across these regions further supports this conclusion.

**(iii)** **Criterion for suppressed WS_2_ on multilayer MoS_2_**
For regions where the MoS_2_ template exceeds several layers (Fig. 5k–m), the analysis focused on whether any additional periodic lattice contrast beyond MoS_2_ is resolvable. In multilayer-template regions where growth is inhibited, only the MoS_2_ lattice periodicity is observed, and no extra lattice features attributable to an overgrown WS_2_ layer are detected under the same imaging conditions, consistent with suppressed WS₂ nucleation in these areas.

**
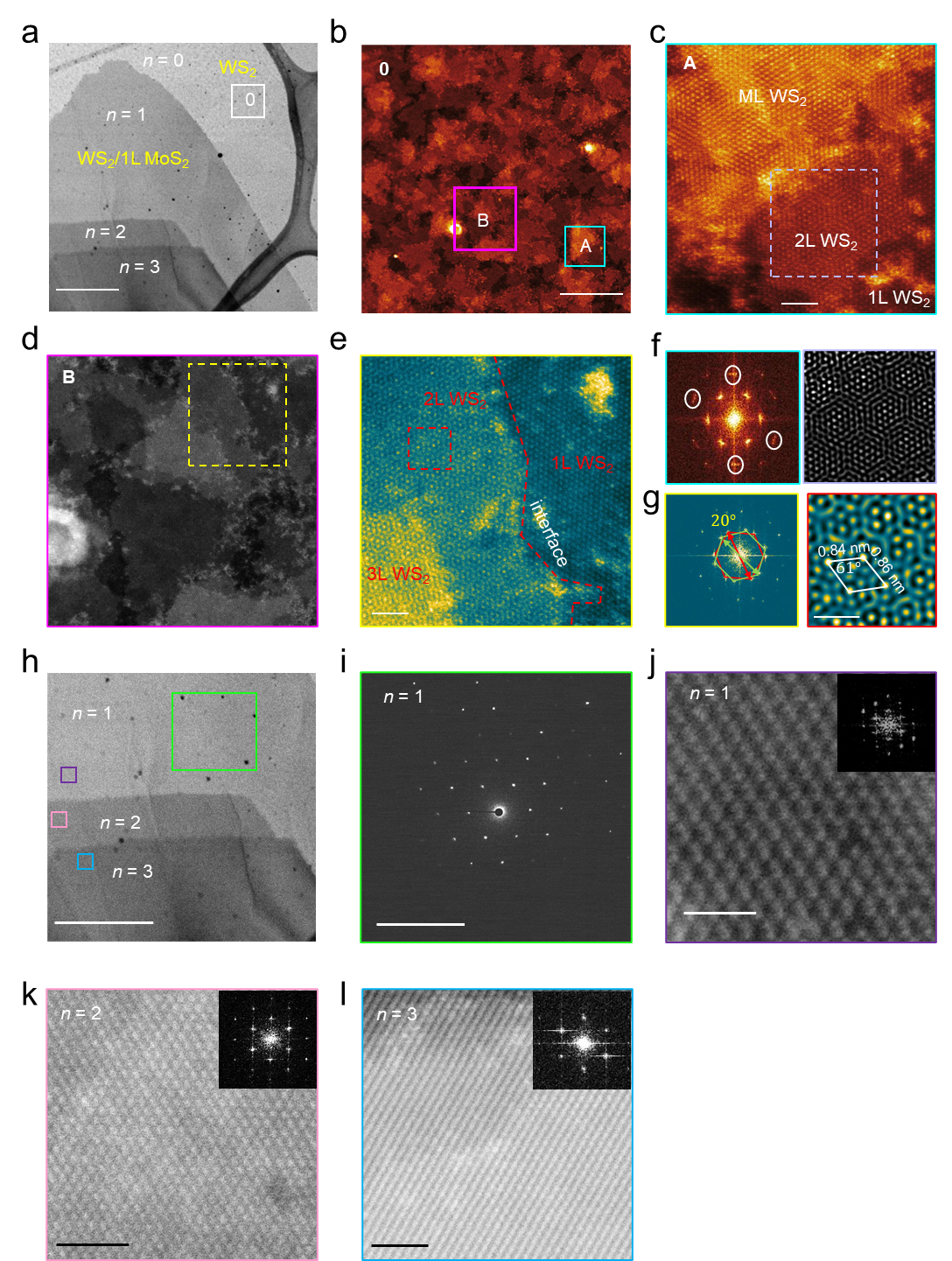
**

**Fig. S9 | Atomic-scale characterization of WS_2_ microstructure in the reciprocal WS_2_-on-MoS_2_ configuration. a,** Low-magnification TEM overview of a representative region containing WS_2_ grown on bare SiO_2_ and on monolayer MoS_2_, with the corresponding areas labeled. **b,** Survey HAADF-STEM image of the WS_2_/SiO_2_ region; boxes A and B mark the locations analyzed in panels **c** and **d**, respectively. **c,** Atomic-resolution image showing local thickness inhomogeneity within the WS_2_/SiO_2_ region (1L, 2L and multilayer WS_2_ patches). **d,** Lower-magnification image of the WS_2_/SiO_2_ region (box C) used for additional analysis. **e,** Atomic-resolution image across a representative thickness interface (dashed line) between 1L and thicker WS_2_ within the SiO_2_-grown region; the red box indicates the area used for the moiré reconstruction in g. f, FFT (left) and masked/filtered reconstruction (right) from the bilayer region in c, revealing rotational misalignment between WS_2_ layers. g, Quantitative moiré analysis of the interface in **e**, where the FFT (left) reveals a ~20° twist angle and the filtered real-space image (right) yields a periodicity of 0.84–0.86 nm. **h,** Low-magnification plan-view TEM survey of the thickness-gradient region for WS_2_ grown on MoS_2_ templates, with the local template layer number labeled from *n* = 1 to *n* = 3. The colored boxes indicate the specific locations characterized in panels **i-l**. **i,** Representative SAED pattern acquired from the monolayer template region (*n* = 1, green box in **h**) showing sharp, discrete hexagonal reflections that confirm a well-defined in-plane registry locked to the template. **j–l,** Atomic-resolution HAADF-STEM images and corresponding FFTs (insets) acquired from regions with template thicknesses of *n* = 1 (purple box, **j**), *n* = 2 (pink box, **k**), and n = 3 (blue box, **l**). The consistent observation of a single set of hexagonal diffraction spots in the FFTs across the entire *n* = 1–3 range demonstrates that the epitaxial registry is robustly maintained and undisturbed by local variations in the underlying template thickness. Scale bars: 500 nm (a,h); 50 nm (b); 2 nm (c,e,k,l) ; 10 nm (d); 1 nm (g,j). 10 nm^-1^ (i),

**5.3. Cross-sectional verification of selective growth**

To rigorously validate the vertical layer structure and the "active" growth window identified by Raman mapping, we performed cross-sectional scanning transmission electron microscopy (STEM) and energy-dispersive X-ray spectroscopy (EDS) on three representative regions.

- **Homo-stacking on SiO_2_** (Fig. S10a): The cross-section of WS_2_ grown on the bare oxide reveals a bilayer structure. Both the top and bottom layers exhibit similar dark contrast in the bright-field (BF) image, consistent with homo-compositional W–W stacking where both layers have the same high atomic number ($Z_{W}=74$).
- **Hetero-stacking on 1L MoS_2_** (Fig. S10b): In the monolayer template region, the heterostructure is clearly resolved. The BF-STEM image shows a distinct Z-contrast difference: the upper layer appears darker (W-rich WS_2_), while the lower layer is lighter (Mo-rich MoS_2_, $Z_{Mo}=42$). This confirms the formation of a vertically stacked WS_2_/1L-MoS_2_ heterobilayer with an atomically sharp interface.
- **Suppression on MoS_2_** (Fig. S10c): In contrast to the active growth on thin templates, the cross-section from the thick-template region reveals only the MoS_2_ multilayer stack. No additional layer attributable to WS_2_ is observed at the surface. This provides direct microstructural evidence for the "shut-off" mechanism on multilayer template, consistent with the suppression observed in Raman mapping.
- **Chemical Validation (EDS)** (Fig. S10d): To corroborate these structural assignments, EDS elemental mapping was performed. The Tungsten (W) signal is strictly confined to the top layer, verifying the formation of a discrete overlayer. It should be noted that the molybdenum (Mo) and sulfur (S) signals appear spatially correlated in the underlying layers. This is due to a spectroscopic overlap between the Sulfur K-alpha line (2.307 keV) and the Molybdenum L-alpha line (2.293 keV), which cannot be fully resolved.
- **Structural Continuity** (Fig. S10a–c): Within the inspected cross-sectional fields of view, the TMDC template layer appears structurally continuous without obvious open channels that would expose the substrate, providing additional evidence against pinhole-mediated epitaxy.

**
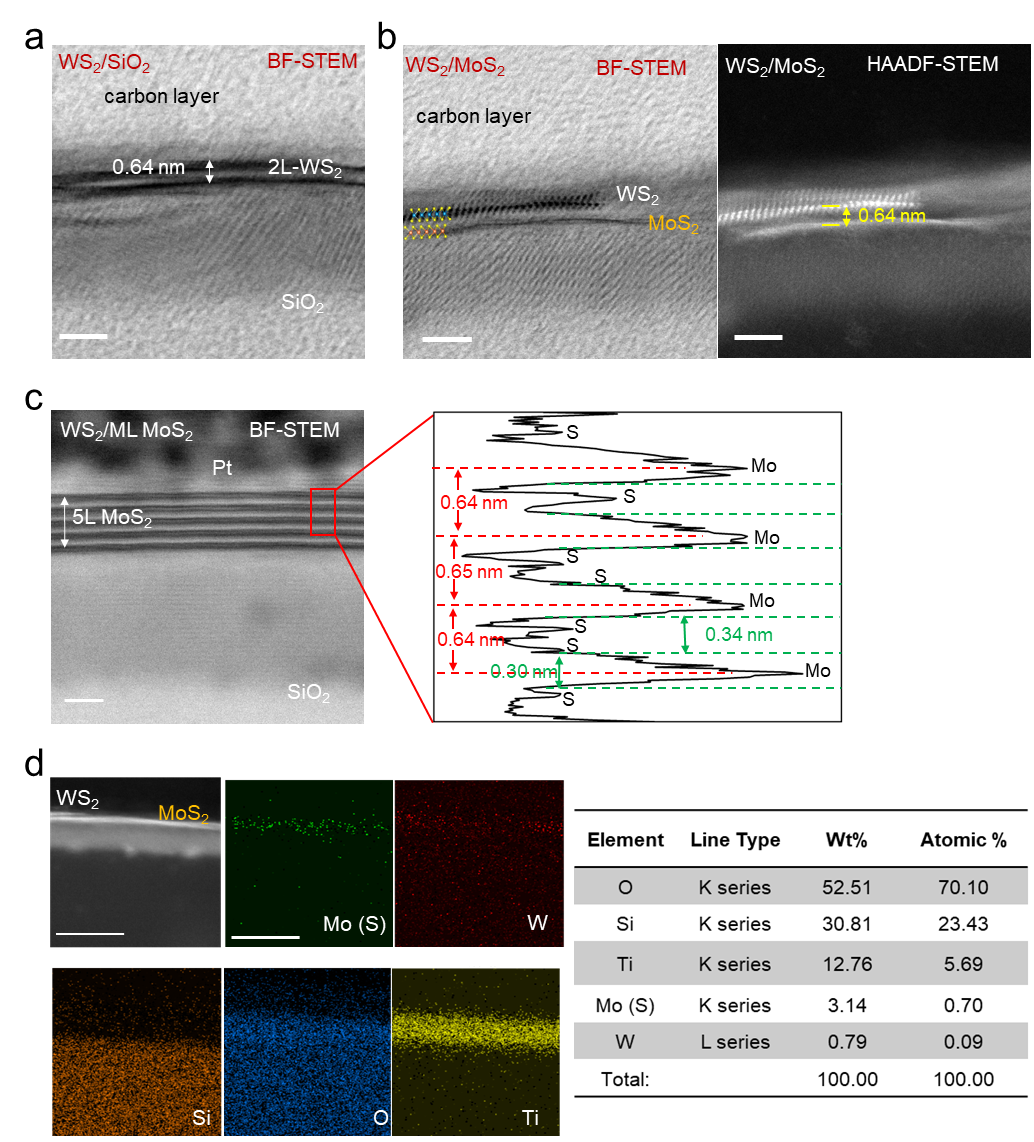
**

**Fig. S10 | Cross-sectional STEM and EDS analysis of thickness-dependent growth selectivity.** **a,** Cross-sectional bright-field (BF)-STEM image of WS_2_ grown on bare SiO_2_, showing a bilayer structure with uniform contrast consistent with WS_2_ homo-stacking. **b,** Cross-sectional BF-STEM of the WS_2_/1L-MoS_2_ region. The distinct Z-contrast difference between the darker top layer (WS_2_, high Z) and the lighter bottom layer (MoS_2_, lower Z) confirms the formation of a sharp hetero-stacking. **c,** Cross-sectional BF-STEM of the multilayer MoS_2_ region. The absence of any top layer confirms the effective suppression (shut-off) of WS_2_ nucleation on thick templates. **d,** EDS elemental maps showing the spatial distribution of W, Mo, and S. Note that the apparent overlap of Mo and S signals in the template region is due to the energetic coincidence of the Mo L-alpha and S K-alpha peaks. Scale bars: 2 nm (a–c) and 10 nm (d).

**Supplementary Note 6. Multislice HAADF-STEM Simulations**

**6.1. Simulation Methodology**

To validate the vertical stacking order of the MoS_2_/WS_2_ (WS_2_/MoS_2_) heterostructures, we performed multislice STEM simulations using the Dr. Probe software package [2]. The simulation parameters were calibrated to the experimental imaging conditions: an acceleration voltage (U_acc_) of 80.00 kV, a probe convergence semi-angle (α) of 20.0 mrad, and a High-Angle Annular Dark-Field (HAADF) detector range of 60.0–200.0 mrad. The supercell was discretized into slices along the Z-axis to model the electron wave propagation through the atomic layers.

**6.2. Comparative Analysis: Dynamical Scattering and Contrast Modulation**

We performed reciprocal stacking simulations to determine how the "Electron Beam View"—the sequential order of atomic species encountered by the probe—dictates the HAADF-STEM intensity profiles. While tungsten (Z=74) possesses a significantly higher scattering cross-section than molybdenum (Z=42), the final experimental contrast is not a simple function of Z^2^ but is instead governed by the modulation of the electron probe as it propagates through the heterostructure.

- **Case 1: MoS_2_ on WS_2_ (W at the Bottom/Detector Side):** In this configuration, the lighter MoS_2_ layer acts as the initial scattering medium. Due to the high transmissivity of the Mo columns, the incident probe remains well-collimated as it reaches the second layer. Crucially, the sulfur (S) columns situated directly above the tungsten sites act as an electrostatic "lens," facilitating a localized “channeling effect”. This effect converges the electron density onto the heavy W nuclei, resulting in an exceptionally high scattering yield and maximized relative contrast (extremely bright W vs. relatively dark Mo).
- **Case 2: WS_2_ on MoS_2_ (W at the Top/Beam Side):** When the heavy W atoms occupy the first slice, they act as the primary scattering centers. While the W columns appear predictably bright, they also induce significant beam broadening before the probe reaches the underlying template. In this sequence, the Mo atoms at the bottom now benefit from the channeling effect induced by the sulfur atoms in the top WS_2_ layer. This focalization enhances the Mo intensity beyond its intrinsic Z-contrast value, leading to a reduced relative contrast (Bright W vs. Enhanced Mo).

**6.3. Direct Verification of Atomic Column Assignments via Vertical Stacking Analysis**

To verify the atomic identity and vertical positioning of our heterostructures, we performed a quantitative comparison between experimental intensity profiles and **Dr. Probe** multislice simulations.

**a. Atomic Column Assignment in MoS_2_ on 1L-WS_2_**

Representative atomic-resolution HAADF-STEM imaging and corresponding line profiles across a MoS_2_ on 1L-WS_2_ region (Fig. S11a,b) reveal a distinct periodicity in normalized intensity:

- **Top-Layer Mo Interaction:** In this stacking sequence, the incident electron beam first encounters the top MoS_2_ layer. As the Mo atoms (Z=42) are the primary points of contact for the probe, their HAADF intensity remains relatively low compared to the heavy atoms beneath them.
- **Sulfur-Mediated Channeling:** The sulfur (S) atoms in the topmost layer act as an electrostatic "lens," facilitating a strong channeling effect. This effect focuses the electron probe into the bottom WS_2_ layer, thereby enhancing the scattering yield from the W columns and producing the maximized contrast fingerprint.
- **Experimental Confirmation:** As observed in the experimental line profile (Fig. S11b), the W columns appear significantly brighter than the Mo columns. This result is perfectly reproduced in the multislice simulation (Fig. S11c), confirming that the maximized relative intensity of W is a direct consequence of its position at the bottom (detector side) of the heterostructure.

**b. Atomic Column Assignment in 1L-WS_2_ (Reference Case)**

To further validate our assignment, we compared the heterostructure profile with a simulated reference of a WS_2_ monolayer (Fig. S11d). This comparison highlights the structural differences between the two systems:

- Absence of Mo Signature: In the 1L-WS_2_ profile, the intermediate peaks corresponding to the Mo atomic columns are absent.
- S-Column Contrast: The line profile for 1L-WS_2_ exhibits a dominant peak for Tungsten (W) and a significantly lower intensity peak for the Sulfur (S) columns.
- Conclusion: The distinct difference between the simulated 1L-WS_2_ profile (Fig. S11d) and our experimental heterostructure profile (Fig. S11b) confirms that the *n* = 1 region is a vertically stacked MoS_2_/1L WS_2_.

**c. Atomic Column Assignment in MoS_2_ on 2L WS_2_ (*n* = 2)**

- Building on the ***n* = 1** analysis and the “electron-beam view” concept discussed above, we applied the same workflow—(i) extracting a normalized HAADF line profile from the experimentally labeled *n* = 2 region and (ii) comparing it with multislice simulations based on the corresponding stacking models—to determine whether the region is a vertically stacked MoS_2_/2L-WS_2_ heterostructure.
- Effect of increasing template thickness (*n* = 2L):
  Introducing a second WS_2_ layer increases dynamical scattering, making the HAADF contrast more sensitive to the vertical encounter order of the atomic layers. Consequently, the profile becomes more strongly W-dominated, while Mo-related contributions may appear as weaker shoulders/intermediate features rather than prominent peaks.
- Experimental fingerprint in the *n* = 2 region:
  The experimental normalized profile shows (i) pronounced high-intensity maxima consistent with W-containing columns and (ii) reproducible intermediate modulation between neighboring W maxima. The presence of this additional modulation indicates that the signal cannot be explained by WS_2_ thickness contrast alone.


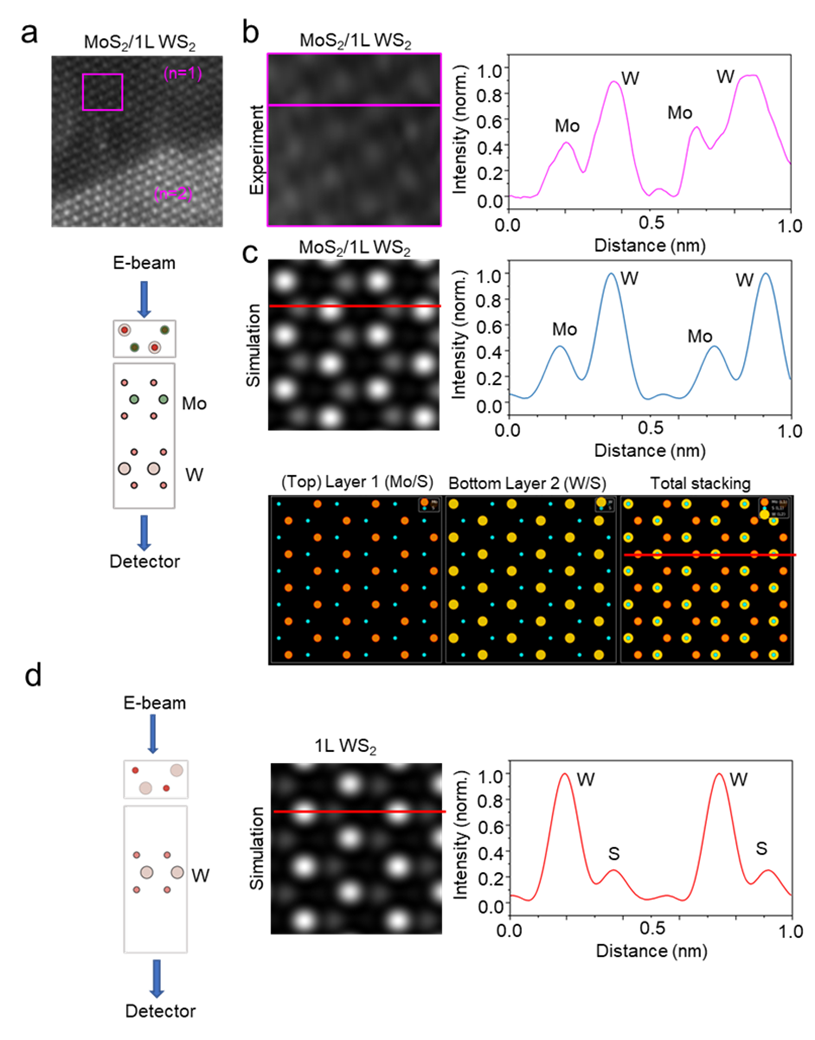


**Fig. S11 | Identifying Atomic Column Stacking through Simulation Comparison. a,** Survey HAADF-STEM image containing regions labeled as MoS_2_ on 1L-WS_2_ (*n* = 1) and MoS_2_ on 2L-WS_2_ (*n* = 2); the magenta box indicates the *n* = 1 area analyzed in this figure. **b,** Atomic-resolution HAADF-STEM image from the *n* = 1 region and the corresponding normalized intensity line profile extracted along the indicated line, highlighting the periodic modulation of metal- and chalcogen-column intensities. **c,** Heterostructure Simulation (MoS_2_ on 1L WS_2_): Multislice simulation confirming that the MoS_2_ layer resides on top of the WS_2_ template. The profile matches experimental data because the top-layer sulfur (S) columns focus the electron beam directly onto the underlying W atoms via a channeling effect, yielding "maximized contrast" where W peaks are significantly dominant. **d,** 1L WS_2_ reference simulation: Simulated comparison of a bare WS_2_ monolayer. The profile exhibits a simple alternating pattern of W and S peaks, lacking the characteristic Mo signal and the specific peak broadening found in the experimental heterostructure, confirming the vertical growth of MoS_2_ on the WS_2_ template.


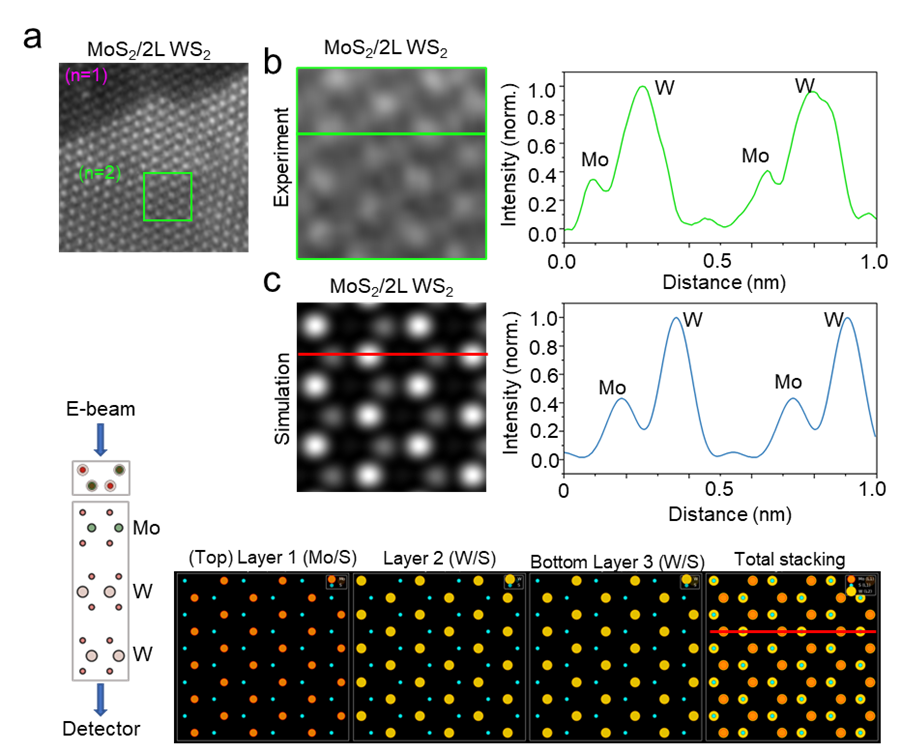


**Fig. S12 | Multislice HAADF-STEM simulation for atomic-column assignment in the MoS_2_ on 2L-WS_2_ region (*n* = 2). a,** Survey HAADF-STEM image showing regions labeled *n* = 1 and *n* = 2; the green box marks the *n* = 2 area analyzed here. **b,** Experimental atomic-resolution HAADF-STEM image from the *n* = 2 region and the corresponding normalized intensity line profile extracted along the indicated line (green). **c,** Dr. Probe multislice simulation based on the MoS_2_/2L-WS_2_ stacking model, with the simulated HAADF image and line profile (blue) extracted along the same direction (red line). The atomic model schematics (bottom) depict the projected column positions for the individual layers and the combined stacking (color-coded by species), illustrating the vertical sequence used in the simulation. The agreement in peak sequence and relative intensity hierarchy supports assignment of the *n* = 2 region as a vertically stacked MoS_2_ on 2L-WS**_2_** heterostructure.

**d. Atomic Column Assignment in WS_2_ on 1L WS_2_ (*n* = 1)**

For the WS_2_/1L-MoS_2_ stacking, the probe interacts first with the top W sublattice, so a substantial portion of the high-angle scattering is generated in the entrance layer. This strong initial interaction also redistributes the probe intensity as it propagates to the lower MoS_2_ layer (i.e., the probe arriving at the second layer is no longer identical to the incident probe).

As the transmitted wave continues through the stack, the periodic potential of the top-layer chalcogen framework guides part of the remaining intensity into the projected columns beneath it. When the lower Mo columns are aligned (or near-aligned) under these sites, the Mo columns can receive enhanced local illumination compared with an isolated MoS_2_ monolayer, increasing the Mo-related peak height in the HAADF line profile.

Net consequence: W peaks remain strong (dominant entrance-layer scattering), but the Mo peaks are partially enhanced, yielding a compressed W–Mo intensity separation relative to the opposite vertical order. This stacking-specific redistribution of intensity provides the physical basis for using multislice simulations to discriminate the vertical sequence in the WS_2_/1L-MoS_2_ region.

**
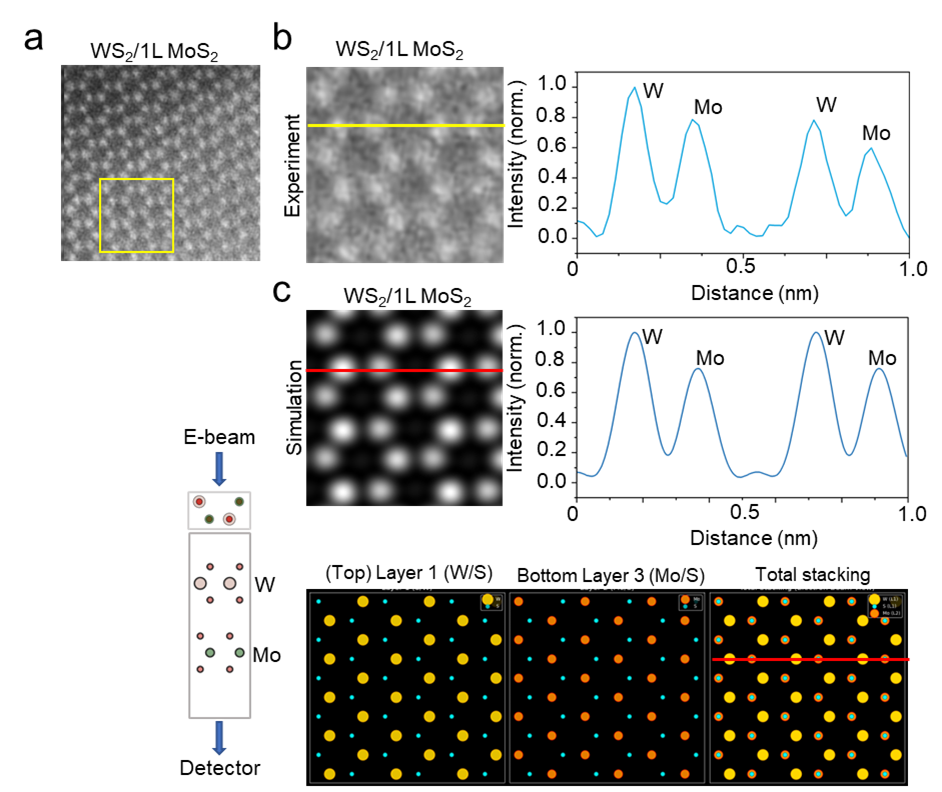
**

**Fig. S13 | Identifying atomic-column stacking in WS_2_/1L-MoS_2_ through HAADF-STEM multislice simulation.** a, Survey HAADF-STEM image; the yellow box marks the heterostructure area analyzed in this figure. **b**, Atomic-resolution HAADF-STEM image from the selected region and the corresponding normalized intensity line profile extracted along the indicated line, showing the periodic modulation of projected atomic columns. **c**, Heterostructure simulation (WS_2_ on 1L-MoS_2_): Heterostructure simulation (WS_2_ on 1L-MoS_2_): Multislice simulation based on the WS_2_/MoS_2_ stacking model reproducing the experimental peak sequence. With W located in the entrance (top) layer, the simulated profile shows dominant W maxima together with a non-negligible Mo contribution from the underlying MoS_2_ layer, confirming that the relative W/Mo peak amplitudes depend on the vertical stacking sequence.

**References**

1. A. Laturia, M. L. Van de Put, W. G. Vandenberghe. Dielectric properties of hexagonal boron nitride and transition metal dichalcogenides: From monolayer to bulk. npj 2D Materials and Applications. **2**(1), 6 (2018). <https://doi.org/10.1038/s41699-018-0050-x>

2. J. Barthel. Dr. Probe: A software for high-resolution stem image simulation. Ultramicroscopy. **193**, 1–11 (2018). <https://doi.org/10.1016/j.ultramic.2018.06.003>
